# Encoded Cell‐Material Interactions to Reroute Cytokine Signaling for Regenerative Medicine

**DOI:** 10.1002/adhm.71481

**Published:** 2026-07-28

**Authors:** Zachary M. Eidman, Jhanvi Sharma, Joanne C. Lee, Nicholas F. Schulze, Hannah J. Brien, Kevin C. Corn, Marjan Rafat, Jonathan M. Brunger

**Affiliations:** ^1^ Department of Biomedical Engineering Vanderbilt University Nashville Tennessee USA; ^2^ Department of Chemical and Biomolecular Engineering Vanderbilt University Nashville Tennessee USA; ^3^ Department of Radiation Oncology Vanderbilt University Medical Center Nashville Tennessee USA

**Keywords:** bioactive materials, cell‐based therapy, regenerative engineering, synthetic Notch

## Abstract

Regenerative engineering harnesses materials science and stem cell biology to develop strategies to repair damaged and diseased tissue. Despite advances in designer materials, few techniques effectively provide auto‐regulated feedback mechanisms that govern how cells sense and respond to discrete microenvironmental changes. Here, we demonstrate that the artificial, juxtacrine‐like receptor synthetic Notch (synNotch) can be activated by endogenous multimeric cytokines in solution, without immobilizing materials, revealing a previously unreported activation modality and yielding up to 24‐fold dynamic range. To broaden synNotch sensing to monomeric cytokines, we developed nMATRIX, a co‐engineered material‐cell platform that detects endogenous, soluble ligands and routes them to programmed gene circuits with spatially confined effects. nMATRIX can be tuned to recognize the interleukins IL‐1β and IL‐6 using synNotch receptors plus cognate biomaterials, yielding more than 68‐fold dynamic range and converting these inflammatory inputs into orthogonal outputs that reprogram nearby cell phenotypes. nMATRIX functions across multiple cell types and can incorporate the synNotch‐related SNIPR synthetic receptor platform. nMATRIX repurposed inflammatory signals and converted them into anti‐inflammatory cues to modulate macrophage surface marker expression. Thus, nMATRIX couples native soluble cues to customized cellular responses with tunable sensitivity, offering a flexible materials‐based approach for self‐regulating regenerative therapies.

## Graphical abstract

We present native MATRIX (Material Activated To Regulate Inducible gene eXpression), a co‐engineered material‐cell platform. Surfaces functionalized with antibodies for endogenous soluble ligands (e.g., IL‐1β or IL‐6) capture and immobilize ligands for presentation to a cognate, engineered synthetic Notch receptor, releasing a transcription factor (TF) to activate programmable, regenerative transgene expression in engineered cells.

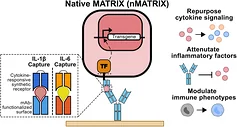

## 1 Introduction

Regenerative engineering integrates modern medicine with engineering principles to treat a variety of conditions including injuries, chronic diseases, and age‐related degeneration [1]. Recent advances in the areas of biomaterials, stem cell biology, and tissue engineering have enabled development of therapies that can repair and regenerate damaged tissue [2, 3]. Despite this progress, a number of barriers have prevented these therapies from achieving clinical translation, including immune rejection, imprecise therapeutic control, and manufacturing scalability [4]. In chronic inflammatory diseases such as rheumatoid arthritis (RA), immune dysregulation in diseased tissue further complicates the adoption of new therapies [5, 6]. Furthermore, current therapeutic platforms lack the capacity to sense and respond to fluctuating biomolecular and mechanical cues, especially in the context of inflammation, which relies on distinct cues to achieve resolution [7, 8, 9, 10]. This underscores a pressing need for a new class of auto‐regulated and adaptive platforms that can sense dynamic environmental signals and provide context‐specific, therapeutic responses to re‐establish tissue homeostasis.

Recent developments in materials science have strengthened regenerative engineering efforts by providing new biomaterials that adapt to diverse inputs to coordinate conditional behaviors of cells. These include materials responsive to niche factors such as redox conditions, proteases, temperature, and the biomechanics of the local environment [11, 12, 13, 14, 15, 16, 17, 18, 19, 20, 21, 22]. Such materials allow for controlled release of therapeutic agents to elicit favorable cellular responses. Materials have also been developed to adapt based on exogenous inputs, such as light [23, 24, 25, 26], for finely tuned spatiotemporal control over material properties and consequent cell behaviors. While these innovative platforms demonstrate a clear shift toward intelligent therapeutic interfaces, they are restricted to unidirectional modes of engagement; that is, once remodeled by proteases or ROS‐reactivity, or induced to release payloads in response to light or temperature changes, their adaptive features are exhausted, and the materials are unable to continue to instruct cellular behaviors to resolve inflammation or encourage neo‐tissue synthesis.

To overcome limitations associated with material‐based strategies, advances in synthetic biology have opened new avenues for engineering cells as therapeutic agents to complement and augment activities enabled via biomaterials that serve as cell delivery vehicles or scaffolds. A diverse repertoire of artificial receptors allows engineered cells to respond to a range of inputs including small molecules, orthogonal proteins, and endogenous cytokines or growth factors [27, 28, 29, 30, 31, 32, 33, 34, 35, 36, 37]. Recently, we reported a platform referred to as MATRIX (Material Activated To Regulate Inducible gene eXpression) [38], which exploits the synthetic Notch (synNotch) artificial receptor to activate customized transgene expression programs in engineered cells in response to selected inputs presented via functionalized biomaterial surfaces. Similar to native Notch, potent synNotch activation requires ligand immobilization; thus, transgene expression in the MATRIX platform is highly localized and constrained to niches decorated with immobilized activators. Our prior work demonstrated the ability to leverage the MATRIX system to allow engineered cells to respond to soluble, bioinert inputs such as green fluorescent protein (GFP) or mCherry to regulate versatile behaviors relevant to regenerative engineering, including activation of CRISPR‐based transcriptome modifiers, modulation of inflammation, and differentiation of human pluripotent stem cells. While the original MATRIX platform was designed to accommodate well‐characterized, bioinert ligands, this work expands platform capabilities by converting cytokines such as IL‐1 and IL‐6 to MATRIX‐compatible inputs. Here, we develop a method to reroute actions that cells take in response to these native cytokines. This extension, which we refer to as native MATRIX (nMATRIX), is sensitive to lower input concentrations than orthogonal receptor platforms that have previously been reported and can drive activation levels spanning a wide dynamic range in response to such input. We demonstrate the broad utility of this platform, with high functionality in several cell types relevant to musculoskeletal regenerative medicine, including fibroblasts, mesenchymal stromal cells, macrophages, chondrocytes, and synoviocytes. We further show the ability of this platform to divert the outcome of pro‐inflammatory IL‐1β signaling, leading such an input to drive the emergence of anti‐inflammatory M2‐like macrophages rather than the typical, IL‐1‐generated M1‐like pro‐inflammatory phenotype. We also demonstrate effective, material‐independent synNotch signal transduction for distinct native ligand sub‐types via mechanisms that challenge prior reports of synNotch functionality. In doing so, we uncover new rules governing synNotch recognition of native ligands and identify a subset of natural factors that can activate synNotch cells independent of secondary immobilization motifs. Thus, this work informs new strategies for co‐engineering cells and biomaterials with synergistic regenerative medicine functionality, providing new insight into receptor design for engineered cell‐based therapies.

## 2 Results

### 2.1 Expansion of the Matrix Platform to Native Factors

**FIGURE 1:**
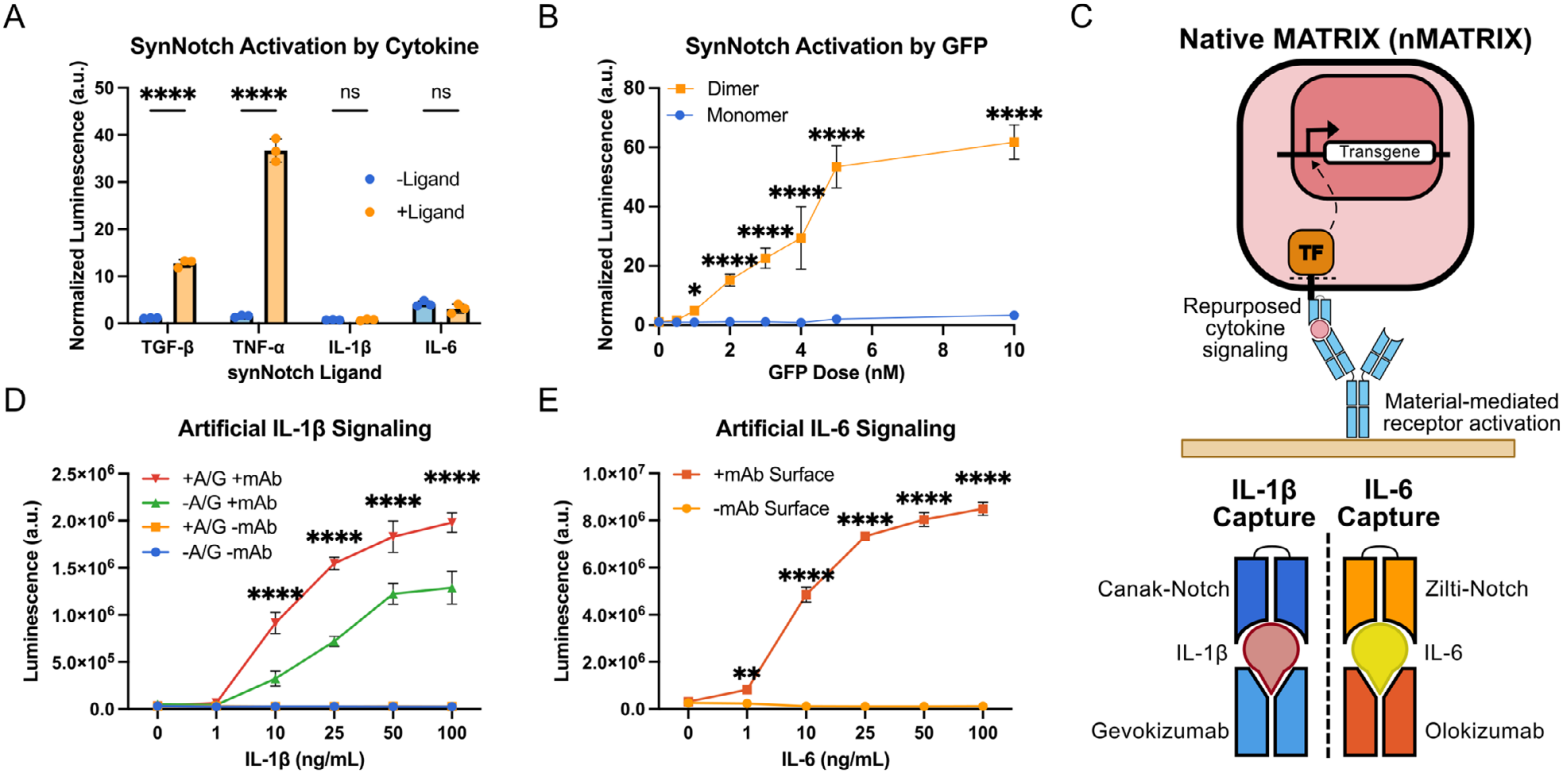
Repurposing MATRIX platform for native inputs. (A) Firefly luminescence normalized to viability indicating activation of synNotch receptors targeted to TGF‐β1, TNF, IL‐1β, and IL‐6. (B) Firefly luminescence normalized to ligand‐free conditions indicating GFP‐induced synNotch activation in response to varied doses of monomer EGFP versus mutant dimer sfGFP. Depicted significance indicated compares +Dimer versus +Monomer at respective dose. (C) Schematic demonstrating native MATRIX system used to immobilize soluble monomeric ligands for presentation to synNotch‐engineered cells. Gevokizumab‐coated surfaces and canakinumab‐synNotch (Canak‐Notch) bind to non‐overlapping epitopes of IL‐1β. Olokizumab‐coated surfaces and ziltivekimab‐synNotch (Zilti‐Notch) cells bind to non‐overlapping epitopes of IL‐6. (D) Firefly luminescence readout in response to varied doses of IL‐1β indicating Canak‐Notch activation in response to Gevo‐surface functionalization with or without Protein A/G. Depicted significance indicates +Protein A/G +mAb group compared to each other group at each respective dose. (E) Dose response of IL‐6‐responsive synNotch when plated on olokizumab‐functionalized surface. Firefly luminescence readout in response to varied doses of IL‐6 indicating Zilti‐Notch activation. Depicted significance compares ‐mAb surface to +mAb surface for each respective dose. All statistical tests were done using a two‐way ANOVA with Tukey's multiple comparisons. *n* = 3 for all experimental groups *p<0.05, **p<0.01, ****p<0.0001.

To develop a platform that enables potent artificial signaling in response to native growth factors and disease‐dysregulated cytokines, we selected a small panel of cytokines including TGF‐β1, TNF, IL‐1β, and IL‐6 as candidates whose signaling could be meaningfully commandeered for regenerative medicine purposes. We derived single chain variable fragments (scFvs) from antibodies previously characterized to bind each of these factors (Table S1). This panel of scFvs was employed to yield a corresponding suite of receptors to allow engineered MSCs to recognize each cytokine as ligand for synNotch. We first screened whether treatment of any of these MSCs with their respective synNotch ligand resulted in receptor activation in the absence of material surface functionalization. We treated the separate synNotch cells with their respective ligand and measured activity of the synNotch‐regulated transgene firefly luciferase. As expected, treatment with soluble IL‐1β and IL‐6 did not activate corresponding synNotch MSCs. However, we were surprised to find that both TGF‐β1 and TNF stimulated artificial signaling in cognate synNotch MSCs to a level of 11‐fold and 24‐fold, respectively, independent of material‐mediated ligand capture and presentation (Figure 1A). Anti‐VEGF synNotch expressing L929 fibroblasts also demonstrated artificial signaling when supplemented with soluble VEGF in the absence of an immobilizing surface (Figure S1).

This finding contradicts prior reports describing synNotch as a juxtacrine‐like signaling channel that requires ligand immobilization to potentiate signaling and that it is non‐responsive to free, soluble inputs [33, 38, 39]. IL‐1β and IL‐6 are like previous inputs we and others have reported on, monomeric signaling factors, while TGFβ‐1 TNF, and VEGF are multimeric ligands (dimer, trimer, and dimer, respectively [40, 41, 42]). Thus, we reasoned that ligand multimerization may enable capture and subsequent presentation to induce synNotch activation independent of biomaterial functionalization. To test this hypothesis, we introduced mutations into superfolder GFP (sfGFP), to adapt it into a dimeric ligand (dGFP) [43]. We then treated GFP‐sensitive, LaG16‐synNotch (LaG16‐Notch) MSCs with a range of concentrations of dGFP or EGFP monomer. Though sfGFP and EGFP have slight differences in sequence, LaG16 has been shown to bind to both efficiently [38, 44]. The 5 nM concentration of dGFP rendered dramatic activation of cells expressing the high‐affinity, LaG16‐Notch receptor, while EGFP did not stimulate substantial signaling at any tested concentration (Figure 1B), consistent with prior reports [28, 38]. We similarly found that dGFP functions as a productive ligand for KOLF2.1J human induced pluripotent stem cells expressing the LaG16‐Notch receptor, suggesting a lack of cell‐type dependency on these findings (Figure S2). These results signify that multimerization converts soluble inputs into those that can productively activate synNotch signaling, while confirming that monomeric inputs such as IL‐1β and IL‐6 require an alternative mechanism of capture and presentation. We thus pursued IL‐1β and IL‐6 as native signaling factors compatible with the MATRIX synthetic signaling paradigm.

To develop a native MATRIX (nMATRIX) platform that can convert IL‐1β or IL‐6 to inputs for customized, orthogonal signaling, we identified reciprocal binding motifs that could anchor each cytokine via epitopes that do not overlap those recognized by a cognate synNotch receptor (Figure 1C). For IL‐1β recognition, we again used IL‐1‐sensitive synNotch cells, engineered to express Canak‐Notch, a synNotch receptor utilizing an scFv derived from canakinumab (Canak), a monoclonal antibody which binds IL‐1β with high affinity (*K*_d_ = 4.1 nM) and is approved for clinical indications such as systemic juvenile idiopathic arthritis, periodic fever syndrome, and gout flares. To develop a functionalized biomaterial surface that can capture and present IL‐1β ligand to Canak‐Notch cells, we identified gevokizumab (Gevo), an investigational IL‐1β inhibitor (*K*_d_ = 0.29 nM), as a candidate motif, as Canak and Gevo had been previously reported to each recognize distinct, 3D discontinuous epitopes of IL‐1β [45]. Thus, to commandeer IL‐1β signaling via nMATRIX, we prepared Gevo‐functionalized surfaces by either passively adsorbing Gevo directly to cell culture wells or by first coating culture surfaces with protein A/G to orient the Fragment antigen‐binding region (Fab) of Gevo away from the culture surface [46]. We then treated cells with IL‐1β concentrations ranging from 1–100 ng/mL. While cells on control, Gevo‐free surfaces exhibited no Canak‐Notch activity when treated with up to 100 ng/ml IL‐1β, we observed potent, dose‐dependent Canak‐Notch activation when cells were cultured on surfaces functionalized with Gevo (Figure 1D). At doses of IL‐1β frequently used in in vitro inflammatory studies (10–100 ng/mL) [47, 48, 49, 50], we show responses ranging between 32‐fold and 68‐fold increase in activity above baseline. As expected, protein A/G‐treated surfaces resulted in enhanced Canak‐Notch activation as compared to surfaces functionalized via passive adsorption of Gevo, with fold changes in the passive adsorption group peaking at 51‐fold change at the 100 ng/mL IL‐1β concentration. Thus, by co‐engineering Canak‐Notch MSCs and a cognate, IL‐1‐capturing surface, we were able to apply the nMATRIX paradigm to enable material‐mediated synthetic signaling in response to a pervasive, pro‐inflammatory cytokine.

We similarly developed an nMATRIX variant designed to rewire cellular responses to IL‐6. We engineered an IL‐6‐specific receptor, Zilti‐Notch, which borrows variable light and heavy chains from ziltivekimab (Zilti), which blocks IL‐6 and IL‐6R classical signaling and is thought to bind to the so‐called Site I of IL‐6 (*K*_d_≤1pM) [51]. We then selected olokizumab (Olok), which binds to Site III of IL‐6 (*K*_d_ = 10 pM), as the surface‐functionalization motif for IL‐6 anchorage and display to Zilti‐Notch cells [52]. AlphaFold‐Multimer modeling of IL‐6, ziltivekimab scFv, and Olok heavy and light chains also predicted the binding of IL‐6 at these sites (Figure S3). Building off our success with IL‐1β recognition, we prepared a protein A/G‐coated surface decorated with Olok (Figure 1C). We again observed robust dose‐ and material‐dependent responses to a native cytokine via the nMATRIX platform, with Zilti‐Notch cells exerting a significant response to levels of IL‐6 across the full range of concentrations tested (1–100 ng/mL) (Figure 1E). At the 10–25 ng/mL concentration range commonly used for in vitro studies that mimic IL‐6‐driven inflammation [53, 54, 55], Zilti‐Notch cells respond with 40‐fold to 63‐fold above background transgene expression. Altogether, these results suggest that the nMATRIX platform can be generalized across monomeric, soluble inputs for which cognate receptor/material recognition motifs can be identified.

### 2.2 Influence of nMATRIX on Cognate Cytokine Bioactivity

**FIGURE 2:**
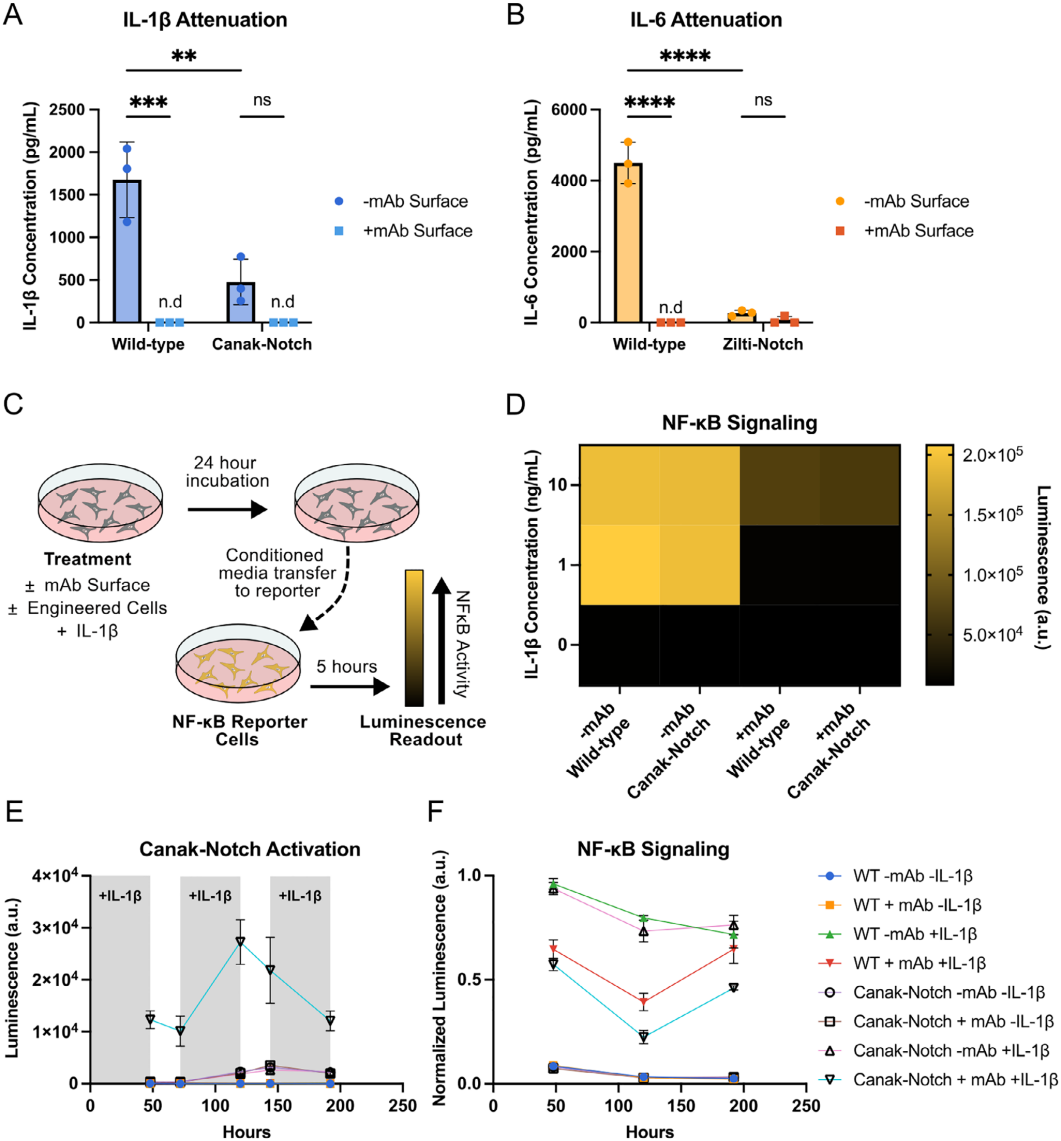
nMATRIX modulates IL‐1β and IL‐6 levels via receptor and surface neutralization. (A) IL‐1β as measured by ELISA 48 h after supplementing 10 ng/mL to either wild‐type mMSCs or Canak‐Notch mMSCs with or without the gevokizumab surface. (B) IL‐6 as measured by ELISA 48 h after supplementing 10 ng/mL to either wild‐type or Zilti‐Notch mMSCs with or without the olokizumab surface. (C) Schematic showing conditioned media treatment of NF‐κB reporter cells. (D) Resultant NF‐κB signaling 5 h after transferring IL‐1β conditioned media to NF‐κB reporter cells. (E,F) nMATRIX is responsive to multiple cycles of cytokine stimulation. (E) Firefly luminescence indicating Canak‐Notch activation in response to repeated doses of IL‐1β. Periods of exogenous 10 ng/mL IL‐1β exposure are indicated (gray shading) at t = 0–48 h, 72–120 h, and 144–192 h, with 24‐h periods of relief at *t* = 48 h and *t* = 120 h. (F) Normalized NF‐κB transcriptional activity 5 h after transferring conditioned media to NF‐κB reporter cells. Signaling is normalized to 10 ng/mL IL‐1β control. All statistics were performed using a two‐way ANOVA with Tukey's multiple comparisons. *n* = 3 for all experimental groups. **p<0.01 ***p<0.001 ****p<0.0001.

Because both the capture motif and the recognition domain of our synNotch receptors consist of neutralizing antibody fragments, we sought to determine the extent to which the nMATRIX platform may attenuate bioactivity of targeted ligands independent of transgene payload production. Thus, we sought to determine how cytokine retention and bioactivity differ in standard cell culture conditions as compared to cell culture conditions modified with nMATRIX constituents (e.g., functionalized surfaces and synNotch‐programmed cells). To do this, we separately treated wild‐type (WT) MSCs or Canak‐Notch MSCs cultured on either control or Gevo‐functionalized surfaces with 10 ng/mL IL‐1β. Forty‐eight hours later, we collected the culture medium and measured the concentration of IL‐1β remaining in the supernatant. Unsurprisingly, we found that Gevo‐functionalization dramatically influenced the amount of IL‐1β recovered from a well, with IL‐1β being undetectable in all supernatants derived from cultures with Gevo‐functionalized surfaces (Figure 2A). We also found that the level of IL‐1β detected in the supernatant of Canak‐Notch cells was significantly lower than IL‐1β detected in WT, Gevo‐free conditions, suggesting that Canak‐Notch cells have an enhanced capacity to capture IL‐1β as compared to control cells. The nMATRIX variant programmed for IL‐6 detection performed similarly, where surface functionalization with IL‐6‐capturing Olok dominated the bioavailability of IL‐6 in supernatant, and Zilti‐Notch expression itself also attenuated the amount of IL‐6 recovered in the supernatant as compared to control MSCs (Figure 2B).

Based on these findings, we investigated the degree to which the IL‐1β‐specific nMATRIX interferes with endogenous cytokine signaling. We programmed a separate line of primary MSCs with an NF‐κB transcriptional reporter that regulates expression of a firefly luciferase. We cultured WT or Canak‐Notch MSCs plated on control or Gevo‐coated surfaces for 24 h in 0, 1, and 10 ng/mL IL‐1β. Cell culture supernatants were then transferred to NF‐κB reporter MSCs for 5 h before the cells were lysed for a luminescence assay (Figure 2C). A subtle but significant reduction in NF‐κB transcriptional activity was observed in recipient cells when culture media were derived from Canak‐Notch versus WT MSCs (Figure 2D). Additionally, the monoclonal antibody surface was unable to completely attenuate NF‐κB signaling in the 10 ng/mL group, indicating native signaling can persist at sufficiently high levels of cytokine input. These results suggest that, while Canak‐Notch cells effectively bind IL‐1β in culture medium, residual cytokine remains highly bioactive. However, consistent with the bioavailability assay, surface Gevo‐functionalization dramatically reduced NF‐κB transcriptional activity in reporter cells treated with IL‐1β conditioned media, indicating that Gevo as employed in these experiments effectively attenuates concentrations of at least 1 ng/ml of IL‐1β.

To determine how nMATRIX responds to repeated cytokine stimulations, we dosed IL‐1β three times at timepoints of 0, 72, and 144 h, while allowing a 24‐h period without exogenous IL‐1β at *t* = 48 h and *t* = 120 h. We evaluated Canak‐Notch activation via a luminescence assay as a readout of inducible firefly luciferase production regulated by Canak‐Notch. Conditioned media from these treatment groups were also applied to NF‐κB reporter mMSCs at the end of each IL‐1β stimulation cycle. Results demonstrate that IL‐1 treatment leads to elevated levels of Canak‐Notch activity after an initial stimulation period of 48 h (Figure 2E). After withdrawal of IL‐1β for 24 h, Canak‐Notch activation levels declined moderately but remained substantially elevated as compared to IL‐1β‐free culture conditions. A second stimulation of Canak‐Notch cells resulted in 2‐fold higher level of activation beyond the original stimulation event after a further 48 h of IL‐1β exposure. Withdrawal of IL‐1β again led to a modest decline in activation, though it did not drop to pre‐stimulation levels. A third 48‐hour stimulation with IL‐1β did not incite a new peak in Canak‐Notch activity; however, output levels remained as high as those observed from the original stimulation cycle. While additional experiments need to be performed to elucidate the factors that enabled the dramatic increase in Canak‐Notch activity during the second stimulation cycle, these results illustrate that the nMATRIX platform can elicit programmable responses to IL‐1β throughout multiple iterative stimulation cycles.

We also observed that IL‐1β treatment induced elevated levels of NF‐κB‐driven luminescence in reporter mMSCs treated with media conditioned from the iterative Canak‐Notch activation assay (Figure 2F). As previously observed, across the first two IL‐1‐stimulation cycles ending at 48‐ and 120‐h, NF‐κB activity levels were attenuated in conditions in which media were collected from groups involving Gevo‐treated surfaces. Interestingly, reporter cells treated with media collected at the end of the third IL‐1 stimulation from WT cells in Gevo‐free conditions exhibited steadily declining NF‐κB activity across the experiment, possibly due to increased anti‐inflammatory factors produced by the immunomodulatory mMSCs. Similar to our prior data, a modest boost in NF‐κB attenuation was conferred when cultures containing Canak‐Notch instead of WT mMSCs served as the source of conditioned media, especially when Canak‐Notch cells were combined with the Gevo‐coated surface. Altogether, these results demonstrate that the nMATRIX platform elements can mitigate durable and cyclically arising inflammatory signals in the absence of synNotch‐driven, anti‐inflammatory transgene expression.

### 2.3 nMATRIX Reroutes Inflammatory Signaling With Orthogonal Outputs in Diverse Cell Types

**FIGURE 3:**
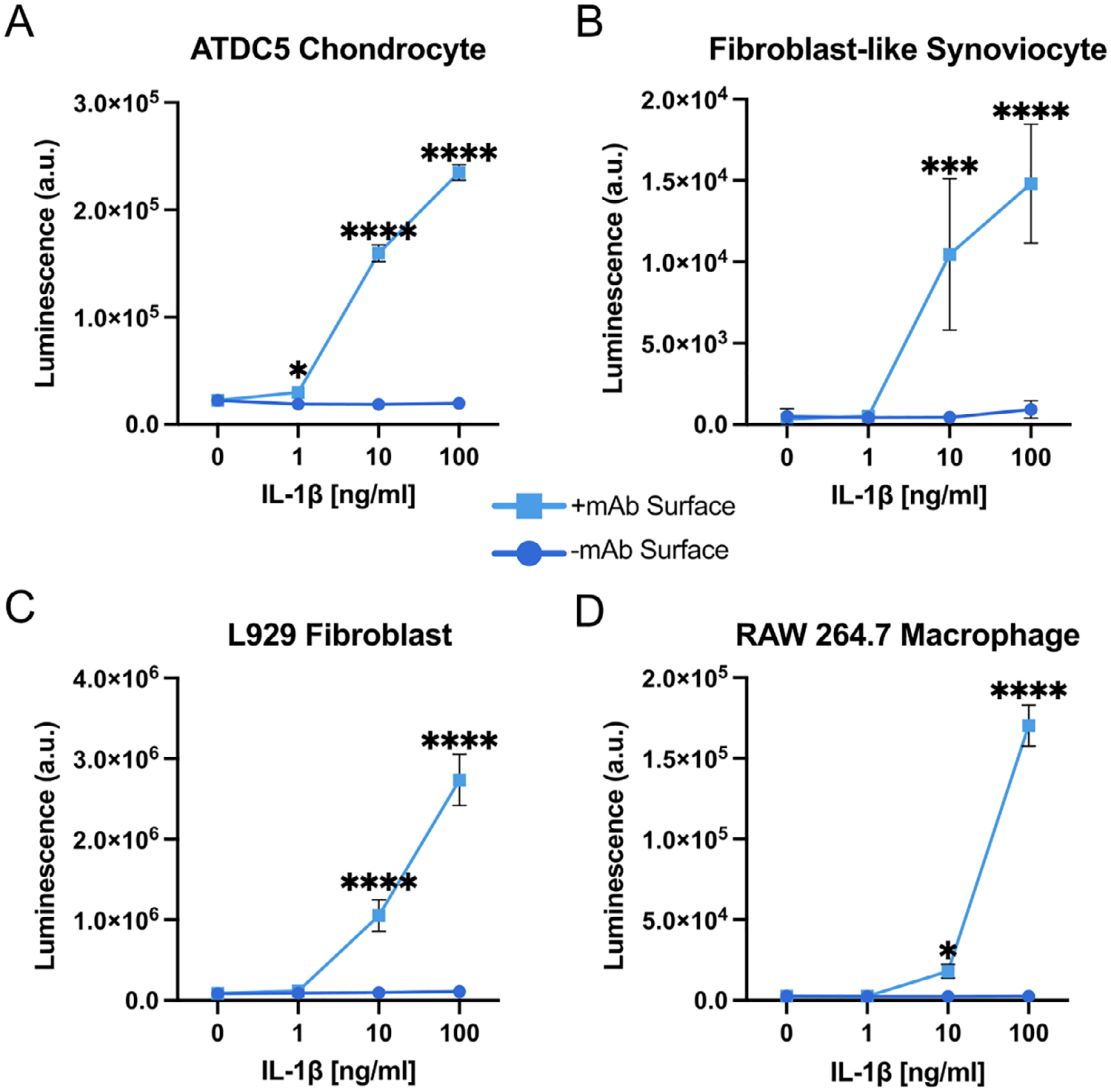
nMATRIX is applicable to different cell types. (A) IL‐1β dose‐dependent activation of (A) ATDC5 chondrocyte‐like cells, (B) primary fibroblast‐like synoviocyte cells, (C) L929 fibroblasts, and (D) RAW264.7 macrophages. All statistics were performed using a two‐way ANOVA with Tukey's multiple comparisons. *n* = 3 for all experimental groups. *p<0.05, ***p<0.001 ****p<0.0001.

We next tested whether the nMATRIX platform is operational in diverse cell types relevant to regenerative engineering. Osteoarthritis, in which inflammatory factors such as IL‐1β and IL‐6 are known to be dysregulated, represents an area of intense study due to the absence of a disease‐modifying therapy. We thus engineered a panel of cells relevant to the homeostasis of articular joints to express the Canak‐Notch receptor (Figure S4) which regulated expression of firefly luciferase and assessed their responsiveness to IL‐1β with and without Gevo‐functionalized surfaces. Previous studies have shown that chondrocytes not only become dysregulated when exposed to proinflammatory cytokines, but they also play an active role in the degradation of cartilage by secreting inflammatory factors [56]. As chondrocytes are also responsible for the production of the cartilage extracellular matrix (ECM), they are an appealing therapeutic target. Indeed, Canak‐Notch chondrocytes exhibited activation with as little as 1 ng/mL of IL‐1β, with more potent activation seen at higher doses and maximal activation of ∼11‐fold over background (Figure 3A). Another pathogenic cell type found in the synovium of arthritic joints includes fibroblast‐like synoviocytes (FLS), which serve as a source of inflammatory cytokines and proteolytic enzymes attributed to joint ECM degradation [57]. Canak‐Notch primary FLS cells showed robust, material‐dependent, synNotch activation at doses of 10 ng/mL and 100 ng/mL (Figure 3B). Canak‐Notch fibroblasts exhibited similar responsiveness to IL‐1β, demonstrating significant synNotch activation compared to those plated in either Gevo‐free or IL‐1‐free conditions (Figure 3C). Lastly, while infiltrating monocyte‐derived macrophages are a hallmark of osteoarthritis and rheumatoid arthritis, prior studies have demonstrated that resident macrophages are a critical modulator in resolving inflammation in affected joints [58, 59, 60]. As such, these tissue macrophages are a prime target for engineering to provide more potent therapeutic responses. As a model for these, we used RAW264.7 cells, an immortalized mouse macrophage cell line. Interestingly, though engineered Canak‐Notch macrophages exhibited a robust, 66‐fold response at the 100 ng/mL IL‐1β concentration, the level of induction upon treatment with 10 ng/mL IL‐1β was modest relative to responses elicited in MSCs, chondrocytes, fibroblasts, and FLS cells (Figure 3D), which may be a result of competition caused by a relatively high expression of the native IL1R1 receptor for IL‐1β in these cells [61]. Altogether, these results indicate that cells known to be both sources of and recipients influenced by IL‐1β can be successfully programmed via the nMATRIX signaling platform for responding to this pro‐inflammatory, arthritis‐driving agent.

### 2.4 Alternative Receptor Chassis for nMATRIX Signal Transduction

**FIGURE 4:**
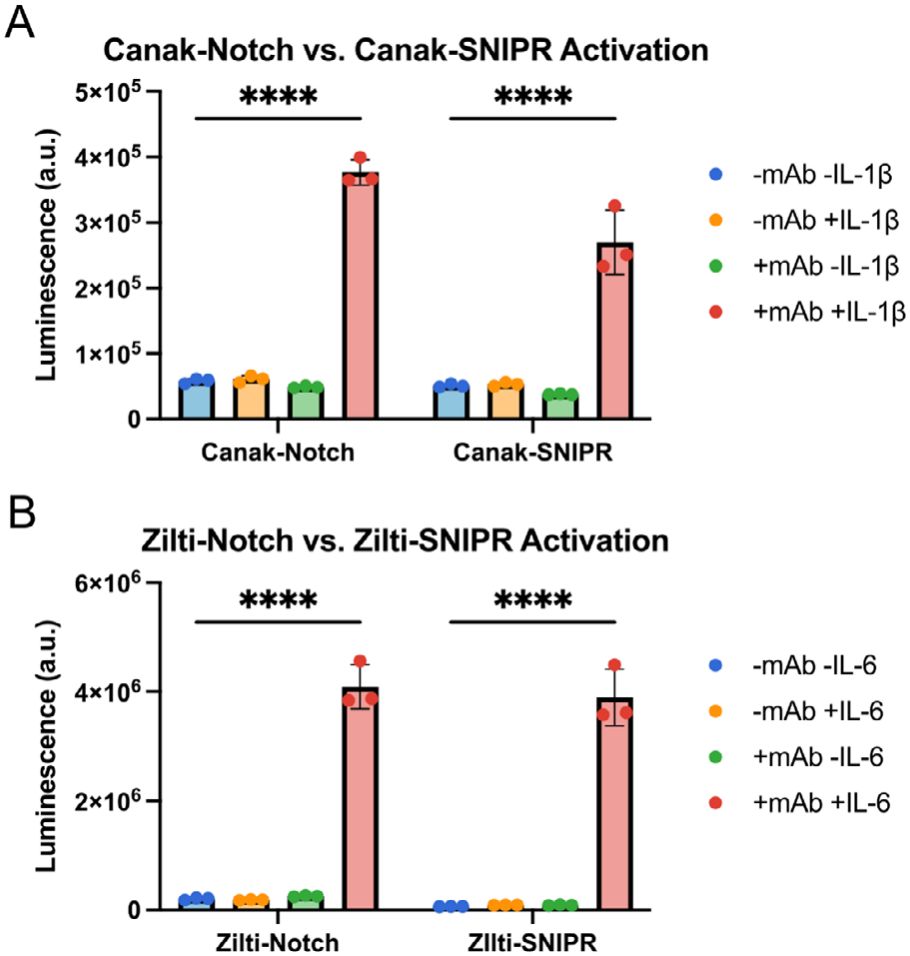
nMATRIX can be adapted to a different receptor platform. (Α) SNIPR engineered with canakinumab scFv can be activated in an nMATRIX‐dependent manner. (Β) SNIPR engineered with ziltivekimab scFv can be activated in an nMATRIX‐dependent manner. All statistics were performed using a two‐way ANOVA with Tukey's multiple comparisons. *n* = 3 for all experimental groups. ****p<0.0001.

The SNIPR platform is an alternative to the classical synNotch regulated intramembrane proteolysis (RIP) receptor [34]. Coding sequences for SNIPRs are smaller than synNotch receptors, making the SNIPR architecture preferred in the context of adeno‐associated virus (AAV) gene delivery vectors. Additionally, recent work illustrated that synNotch and SNIPR activation levels differ in the context of soluble, multimeric inputs: SNIPR‐engineered T cells activated dramatic transgene expression in response to multimeric inputs such as TGF‐β1 due to an endocytic, pH‐dependent mechanism that was not active in synNotch T cells [33]. These factors motivated an assessment of whether nMATRIX is compatible with the SNIPR architecture. Thus, we outfitted MSCs with either Canak‐SNIPR or Zilti‐SNIPR. Results reveal that, despite differences previously reported for SNIPR and synNotch activation mechanisms, the nMATRIX framework accommodates both receptor chassis (Figure 4A,B). As with synNotch cells, SNIPR variants demonstrated dependency on both ligand and surface‐functionalization for induction. While Canak‐Notch activated more potently than Canak‐SNIPR on the Gevo‐treated surface (Figure 4A), Zilti‐SNIPR activated more potently than Zilti‐Notch when compared to their respective ligand‐free and surface‐free groups (Figure 4B), though both receptors had similar maximal activation levels. Thus, while validating the broad utility of the nMATRIX framework for repurposing IL‐1β and IL‐6 as inputs for programmable artificial signaling, these results also suggest that empirical evaluation may be required to determine whether SNIPR or synNotch should be implemented within the nMATRIX paradigm for each selected ligand.

### 2.5 Synthetic Modulation of Macrophage Surface Marker Expression via Native MATRIX

**FIGURE 5:**
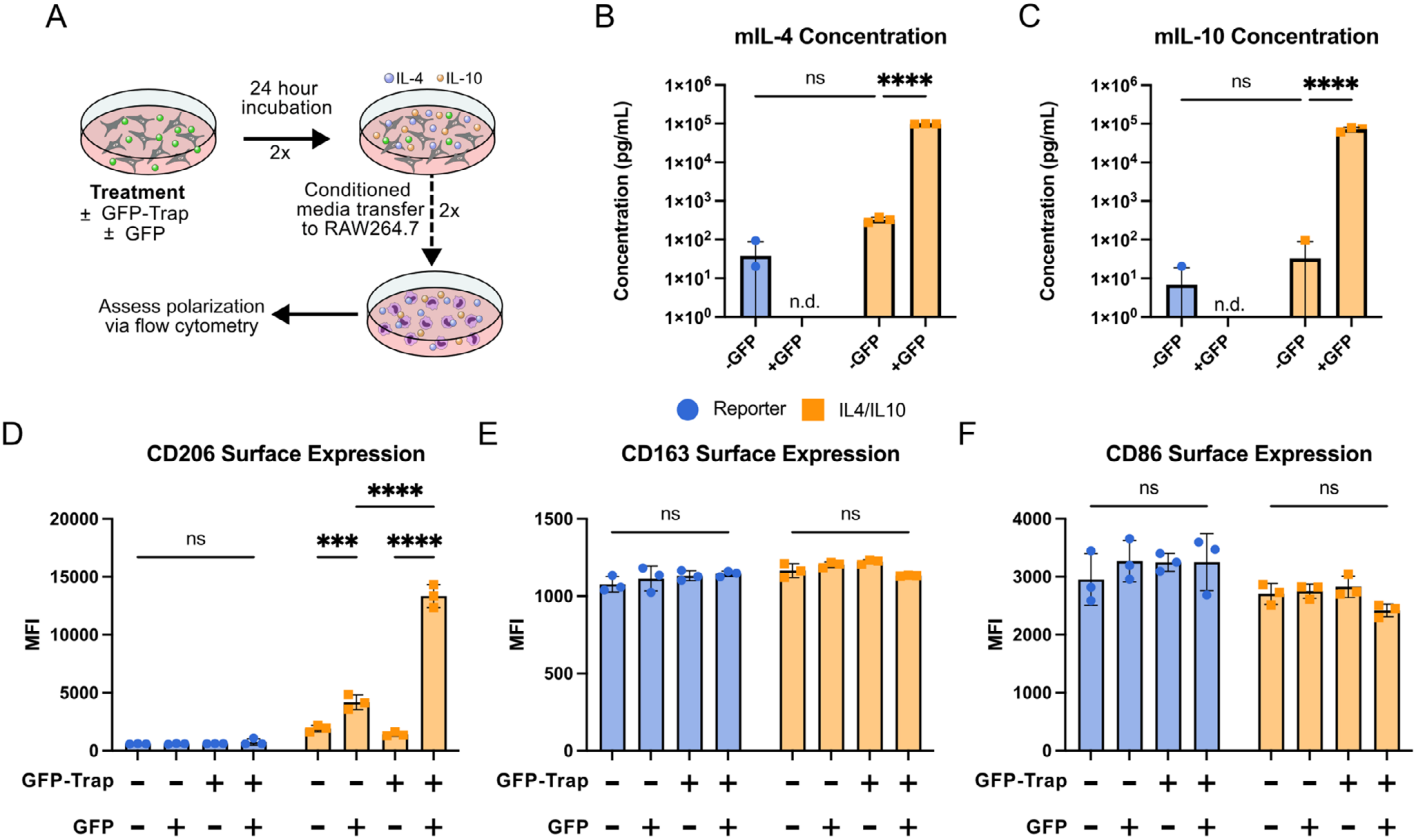
MATRIX can enhance CD206 expression in macrophages. (A) Schematic of experimental setup. GFP‐responsive synNotch mMSCs containing an inducible reporter transgene or an inducible IL4/IL10 cassette were stimulated with GFP. Conditioned media from these cells was added to RAW 264.7 cells at 24 and 48 h timepoints. At 72 h, RAW264.7 surface marker expression was measured via flow cytometry to evaluate polarization. (B), (C) Mouse IL‐4(B) and mouse IL‐10(C) concentrations as measured by ELISA after 24 h of GFP stimulation of anti‐GFP reporter and IL4/IL10 mMSCs. (D)–(F) Quantified mean fluorescence intensity of CD206(D), CD163(E), and CD86(F) surface expression of RAW264.7 cells after treatment with GFP‐induced reporter and IL4/IL10 cell conditioned media. All statistics were performed using a two‐ way ANOVA (B,C) or three‐way ANOVA (D‐F) with Tukey's multiple comparisons *n* = 3 for all experimental groups. ***p<0.001 ****p<0.0001.

Having established the ability to commandeer native cytokines for artificial signaling in diverse cell types, we next turned to evaluate whether the nMATRIX platform can effectively redirect cellular responses to pro‐inflammatory factors to instead execute anti‐inflammatory functions. Due to their association with the pathogenesis and perpetuation of various chronic diseases, we focused on modulating macrophage phenotype. Whereas M1‐like macrophages are considered pro‐inflammatory and contribute to autoimmune diseases and inflammatory disorders, M2‐like macrophages are considered pro‐resolution and aid in restoration of tissue function after injury. A sustained presence of M1‐like macrophages, without a transition to M2‐like phenotypes, underlies the progression of many chronic degenerative conditions. Thus, we sought to determine whether material‐mediated, synNotch‐inducible expression of polarizing factors could evoke emergence of a pro‐regenerative macrophage population as determined by surface marker expression. To disentangle the influence of pro‐inflammatory cytokines on macrophage polarization, we first returned to our orthogonal MATRIX platform, which relies on bioinert signaling factors such as GFP, rather than cytokine, for induction. We thus investigated engineered two LaG16‐Notch MSC lines: one engineered for inducible co‐expression of control reporter genes mCherry and firefly luciferase (Reporter) and another engineered for inducible co‐expression of IL‐4 and IL‐10 (IL4/IL10), which are commonly used to polarize macrophages into M2‐like subtypes [62, 63]. We then plated these GFP‐responsive MSCs onto control surfaces or surfaces functionalized with the GFP‐capturing motif GFP‐Trap (Figure 5A). Conditioned media were collected and added to RAW264.7 cells at 24‐ and 48‐hour timepoints, and the production of IL‐4 and IL‐10 was measured 48 h later via ELISA. While LaG16‐Notch IL‐4/10 MSCs produced minimal levels of IL‐4 or IL‐10 when left untreated, we observed >300‐fold enhancement in both IL‐4 and IL‐10 levels in the LaG16‐Notch IL4/IL10 group when treated with 5 nM GFP (Figure 5B,C). Flow cytometry quantification of macrophage surface markers in macrophages revealed that CD206 was significantly elevated in response to GFP‐induced synNotch expression of IL‐4 and IL‐10 (Figure 5D and Figure S6), indicating an M2a‐like phenotype. CD163 levels were not upregulated in RAW264.7 cells treated with conditioned medium from GFP‐treated LaG16‐Notch IL4/10 MSCs (Figure 5E). Congruent with this, prior reports indicated that inflammatory factors are required to elevate expression of CD163 via IL‐10 signaling [64, 65, 66, 67]. Lastly, MSC culture conditions had little impact on the levels of the M1 marker CD86 (Figure 5F) expressed by macrophages. These results demonstrate that synNotch MSC activity in the context of a MATRIX system can influence macrophage phenotype in a ligand‐, surface‐, and transgene‐dependent manner.

**FIGURE 6:**
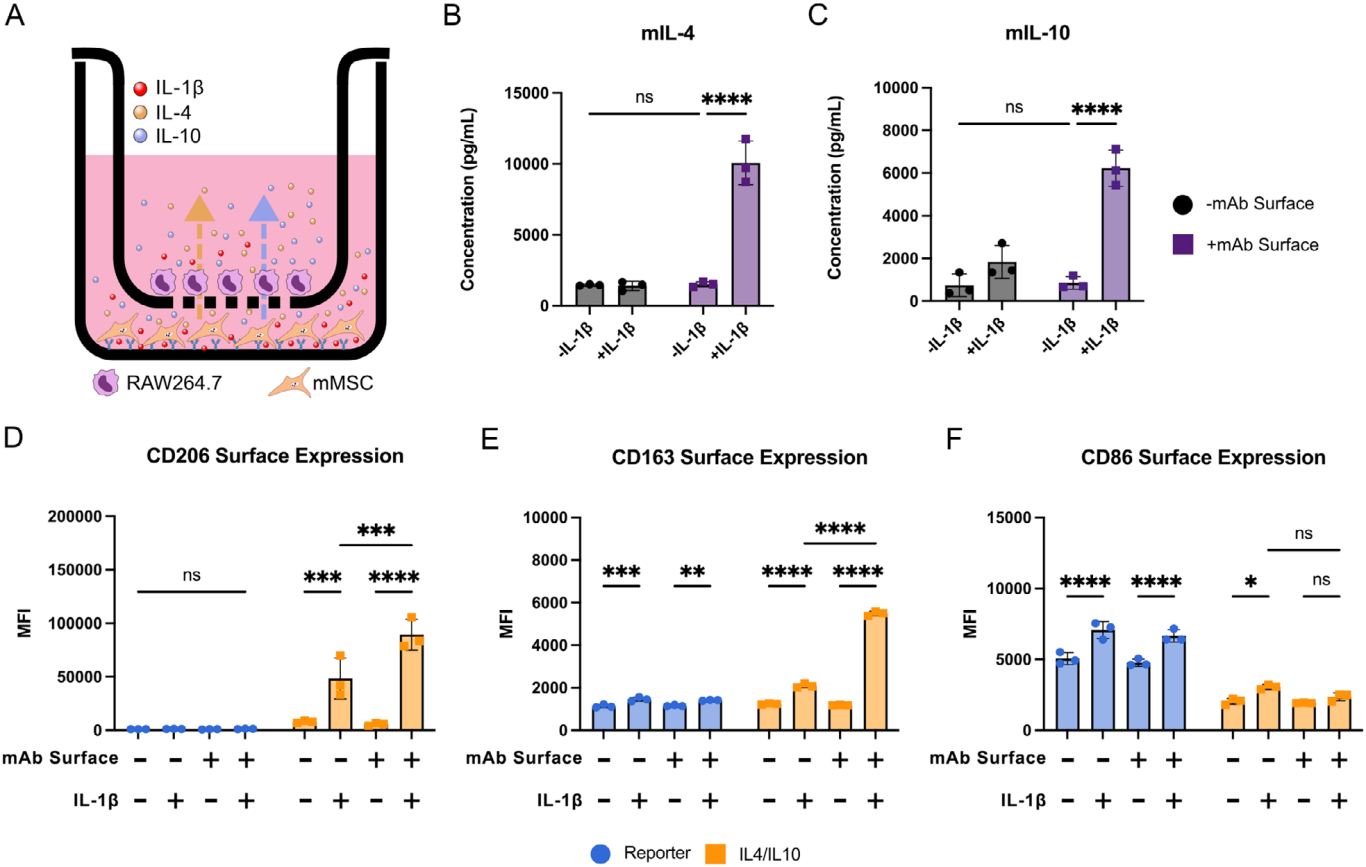
nMATRIX can polarize macrophages toward M2‐like phenotypes. (A) Schematic of Transwell setup. Canak‐Notch mMSCs containing an inducible reporter transgene or an inducible IL4/IL10 cassette were stimulated with 10 ng/mL IL‐1β. After 24 h of mMSC stimulation, RAW264.7 cells in the apical Transwell chamber were transferred to a well with mMSCs in the basal chamber. After 48 h of co‐culture, RAW264.7 surface marker expression was measured via flow cytometry to evaluate polarization. (B, C) Murine IL‐4 (B) and murine IL‐10 (C) concentrations as measured by ELISA after co‐culture with RAW264.7 cells. (D‐F) Quantified mean fluorescence intensity of CD206 (D), CD163 (E), and CD86 (F) surface expression of RAW264.7 cells after treatment with IL‐1β‐induced reporter and IL4/IL10 co‐culture. All statistics were performed using a two‐way ANOVA (B,C) or three‐way ANOVA (D)–(F) with Tukey's multiple comparisons. *n* = 3 for all experimental groups. *p<0.05 **p<0.01 ***p<0.001 ****p<0.0001.

Encouraged by these results, we next interrogated whether the nMATRIX platform could reroute IL‐1 signaling, which would typically drive a CD86_hi_/CD206_lo_/CD163_lo_ expression pattern, to instead render the inverse phenotype of CD86_lo_/CD206_hi_/CD163_hi_ in macrophages. In this experimental configuration, we employed a Transwell co‐culture, where macrophages were cultured in the apical chamber and Reporter or IL4/IL10 Canak‐Notch‐MSCs were cultured in the basal chamber (Figure 6A). We stimulated cells with 10 ng/mL IL‐β and again observed marked ligand‐ and surface‐dependent upregulation of IL‐4 and IL‐10 proteins in media collected at day 3 from Canak‐Notch MSCs (Figure 6B,C). Flow cytometric results revealed that macrophage phenotype was dramatically influenced by each of surface functionalization, IL‐1β treatment, and Canak‐Notch MSC transgene payload (Figure 6D–F and Figure S7). Regardless of the presence of the IL‐1β neutralizing Canak‐Notch receptor and surface‐functionalized Gevo motifs, macrophage CD86 levels were elevated after IL‐1β treatment in the presence of Reporter Canak‐Notch MSCs. Further, CD206 expression was absent, and CD163 levels remained low in co‐cultures with Reporter Canak‐Notch MSCs. In contrast, IL‐1β treatment in co‐cultures with IL4/IL10 Canak‐Notch MSCs resulted in slight but significant elevation of CD86 only in the synNotch‐off, Gevo‐free state. When Gevo‐functionalization enables ligand‐dependent production of IL‐4 and IL‐10 by Canak‐Notch MSCs, CD86 levels are completely attenuated. As in the prior GFP MATRIX experiment, we observed elevated levels of CD206 in response to synNotch‐driven IL‐4 and IL‐10 production, despite the presence of pro‐inflammatory IL‐1β. Furthermore, in the presence of IL‐1β and resultant payload transgenes from IL4/10 Canak‐Notch MSCs, we observed dramatically enhanced CD163 expression. Taken together, these results illustrate that the nMATRIX platform with Canak‐Notch MSCs can be used to reroute the pro‐inflammatory IL‐1β signaling to drive macrophage polarization in alternative directions using pre‐defined, transgene outputs.

## 3 Discussion

Here, we demonstrate that our previously described MATRIX platform, composed of designer biomaterial surfaces and engineered cells, can be configured to sense and respond to native, soluble molecules. Notably, nMATRIX utilizes customizable, orthogonal synNotch receptors that induce expression of downstream gene circuits, allowing for the conversion of various pro‐inflammatory cytokines into alternative outputs. Our platform enables robust, inducible gene expression with high fold‐change activation within a biologically relevant range of cytokine concentrations [47, 48, 49, 50, 53, 54, 55]. Both the material and receptor components of nMATRIX have demonstrated cytokine neutralizing capabilities, which can be used to modulate endogenous inflammatory signaling. Additionally, nMATRIX acts as a Boolean AND gate, requiring engineered cells, ligand and the immobilizing surface to be present to produce programmed outputs, allowing for spatiotemporally controlled therapeutic delivery. nMATRIX offers broad applicability, as it can be used to produce artificial signaling in a wide variety of cell types implicated in musculoskeletal diseases including mesenchymal stem cells, chondrocytes, macrophages and fibroblast‐like synoviocytes, and can be used with alternative synthetic receptor architectures such as SNIPR. Lastly, we demonstrated nMATRIX can be used to convert the highly inflammatory signal IL‐1β into an anti‐inflammatory output to bias macrophages toward a more anti‐inflammatory phenotype. Overall, nMATRIX is a highly customizable platform that allows for material‐dependent, prescribed cellular programming in response to elevated levels of native factors that contribute to inflammatory diseases.

As the immune system plays a significant role in the progression and chronic dysregulation of inflammatory diseases, environmentally responsive biomaterials, including hydrogels [26, 68, 69, 70, 71] polymeric scaffolds [72, 73, 74], and nanoparticles [75, 76, 77, 78], have been developed to modulate the immune environment by delivering immunomodulatory agents in a regulated manner. Although these technologies initially offer improved control and sustained release of therapeutics, this effect dissipates as the respective material degrades and releases its payload. Engineered cell‐based therapies offer a potential solution for auto‐regulated and autonomous immunomodulation, as cells can be tuned to effectively sense and respond to distinct cues in the microenvironment [79, 80, 81, 82, 83, 84, 85, 86]. Here, we demonstrate that nMATRIX can detect inflammatory cues and respond with anti‐inflammatory cues, influencing the phenotype of nearby cells and pushing them toward a more pro‐resolution fate. Additionally, both components of nMATRIX have cytokine neutralizing capabilities, enhancing the therapeutic potential of the platform beyond cellular outputs. As such, nMATRIX can potentially be used to resolve dysregulation in diseased tissue by combining inflammation suppression and regulated cell feedback to regain tissue homeostasis.

Macrophages are a highly plastic cell type with the ability to change phenotype based on cues in the microenvironment. In rheumatoid arthritis, inflammatory macrophages contribute to both the initial onset and progression of disease through production of cytokines such as TNF, IL‐1β and IL‐6 [87]. As such, repolarization of macrophages has become a focus in potential treatments of numerous diseases in which macrophages are implicated [88]. Previous studies have used hydrogels or polymeric scaffolds with inherent physical properties that drive this M2 polarization [89, 90, 91, 92]. Similarly, mRNA‐, plasmid‐, or protein‐loaded materials [93, 94, 95, 96] have been shown to effectively immunomodulate macrophages by delivering anti‐inflammatory cytokines like IL‐4 and IL‐10. This strategy has also been applied via engineered cells that overexpress anti‐inflammatory cytokines like IL‐10 [97, 98]. Though these studies have shown efficacy in polarizing macrophages toward an anti‐inflammatory phenotype, the platforms function unidirectionally and cannot autonomously sense external stimuli to generate customized responses. Here, we demonstrated nMATRIX can effectively reroute IL‐1β signaling to instead produce IL‐4 and IL‐10 via artificial gene circuits in a material‐ and ligand‐dependent manner, which steered macrophages toward a more regenerative, anti‐inflammatory phenotype. This may provide an increased benefit over the previously described platforms in that nMATRIX is an autoregulated system that responds to dynamic cues in the microenvironment. The modular programmability of nMATRIX suggests the opposite, that is, pushing an M2‐like macrophage toward an M1 phenotype via inducible inflammatory transgene production, could be implemented as well. This would be especially useful in a tumor microenvironment, as various studies have shown polarizing tumor‐associated macrophages using inflammatory cytokines can improve immune response to cancer [99].

A core principle of synthetic biology is developing modular frameworks that can utilize native inputs to drive desired cellular outputs. Early synthetic receptors like chimeric antigen receptors (CARs) fuse elements from the endogenous T cell receptor (TCR) signaling apparatus to synthetic binding domains, enabling activation of T cells in response to selected surface marker exhibition [100]. Although CARs have been shown to effectively activate T cells in response to surface markers such CD19 and HER2 [101, 102], and even a soluble, multimeric factor, TGF‐β [103], their outputs are limited to endogenous TCR signaling pathways. Other approaches including chimeric cytokine receptors (ChR) and synthetic cytokine receptors (SyCyRs) hijack native cytokine receptor signaling pathways [104, 105]. These technologies have shown great promise, but their signaling mechanisms are susceptible to crosstalk and have may not port well into diverse cell types. Another modular receptor system, MESA, has demonstrated artificial, orthogonal signaling utilizing dimerizing ligands such dTomato or VEGF [29, 106]. In this work, we have demonstrated synNotch can also be activated using soluble, multimeric cytokines like TNF, TGF‐β1 and VEGF. This contradicts previous reports suggesting that TGF‐β1 could not be used for effective synNotch activation, indicating a potential cell type dependency for this interaction (e.g., adherent vs. suspension cells) [33]. nMATRIX further adds to the synthetic biology toolbox by enabling synNotch signaling with soluble, monomeric, native inputs like IL‐1β and IL‐6 via biomaterial immobilization. Furthermore, we demonstrated nMATRIX can act as a cytokine converter that can be modulated based on desired application. We also showed that nMATRIX effectively signals with other RIP receptors like SNIPR, suggesting the platform can be used with other juxtacrine receptor formats. Lastly, the dual epitope binding methodology applied in this work could potentially facilitate monomeric signaling in other receptor architectures like MESA and CARs by outfitting them with our cognate scFv pairs.

Although a flexible and effective platform, nMATRIX in its current form has various limitations. As material‐mediated ligand immobilization is required for effective signaling, the system would need to be adapted into a 3D model for in vivo applications. Antibody conjugated hydrogels have been previously described, and would be a strong candidate for delivering both immobilizing antibody and engineered cells in vivo [107, 108]. Additionally, the antibodies described in this work are humanized and have limited cross‐reactivity with murine cytokines, and preclinical trials would need to use humanized IL‐1β or IL‐6 mouse models. Furthermore, synNotch‐based systems are susceptible to basal “leak” of engineered gene circuits, leading to unintended ligand‐independent effects. Additionally, when engineered cells produce secreted cytokines, evaluation of the network level effects on nearby tissues needs to be conducted. Additionally, since nMATRIX requires two affinity motifs that bind to distinct epitopes on the same molecule simultaneously, it would likely be difficult to adapt to poorly characterized molecules with few targeting motifs. We demonstrated that nMATRIX is functional in numerous cell types and can be adapted to detect multiple different inputs; however, clinical adoption will require demonstration that the system functions robustly across cell types of interest derived from numerous human donors. Despite these limitations, nMATRIX remains a highly flexible, modular platform with broad use for cellular programming.

In conclusion, we present the nMATRIX platform, a hybrid system integrating material‐guided signal transduction with computational capabilities of engineered cells. Crucially, the platform confines both sensing and effector activity to biomaterial‐contact regions, minimizing off‐site effects. The platform is also highly modular: both the cells and material surfaces can be programmed to recognize and respond to virtually any selected ligand, enabling precise control over cellular behavior. This adaptability is especially valuable in complex pathological environments such as solid tumors, chronic inflammatory conditions, and degenerative tissues, where coordinated, multi‐cellular intervention is often required. By harnessing the native capabilities of diverse cell types and augmenting them with nMATRIX, it becomes possible to design multi‐layered therapeutic systems capable of sensing and responding to their surroundings with high precision. In summary, our platform provides a robust method of producing auto‐regulated signaling in response to physiological inputs like cytokines.

## 4 Materials and Methods

### 4.1 Plasmids and Cloning

Plasmids were constructed using NEB HiFi DNA Assembly Mix (New England Biolabs) and were subsequently transformed into DH5α competent cells. Cultures were plated on LB Agar + Ampicillin (Sigma) and grown overnight at 37°C. Colonies were picked and validated via whole plasmid sequencing (Plasmidsaurus).

### 4.2 Construct Design

Gene fragments for cloning coding sequences for new recognition motifs or transgenes noted in the following were purchased from Thermo Fisher's GeneArt service. New synNotch receptors were derived by exchanging the mAbCII recognition domain in Addgene 223538 [109] with the coding sequences for affinity domains noted in Table S2. The LaG16‐Notch receptor has been previously reported [38, 110, 111], and contains the anti‐GFP LaG16 nanobody [112] in place of the mAbCII recognition domain in Addgene 223538. Addgene 223539 [109] was used as the Firefly luciferase and mCherry reporter payload vector in these studies. To obtain the IL4/IL10 payload vector, the luciferase and mCherry payloads were replaced with coding sequences for murine IL‐10 and IL‐4. To obtain the Canak‐SNIPR and Zilti‐SNIPR vectors, we replaced the Notch transmembrane domain from respective synNotch receptors with coding sequences for the CD8 Hinge, Notch juxtamembrane domain, and Notch transmembrane domain derived from Addgene 188375 [34]. An additional (G_3_S)_3_ flexible linker sequence was inserted between the C‐terminus of each respective scFv and the N‐terminus of the CD8 hinge region to increase flexibility. The coding sequence for a 6x‐His‐tagged dimeric superfolder GFP [43] was cloned in a pET‐family derived bacterial expression vector. The dimeric superfolder GFP variant contains three mutations to promote disulfide bonding by eliminating two naturally occurring cysteines (C48A and C70A) and introducing a free cysteine (D117C). Plasmids and plasmid sequences are available from Addgene, with corresponding depository numbers provided in Table S2.

### 4.3 Virus Production

Lentivirus was produced by transfecting Lx293T cells (Clontech) with 2 µg of transfer vector, 1.5 µg of pCMV‐dR8.91 packaging vector [113] and 0.6 µg of pMD2.G envelope vector [114] (Addgene #12259) using Lipofectamine 3000 (Thermo Scientific) following manufacturer's instructions. 16 h later, medium was exchanged for fresh DMEM‐High Glucose supplemented with GlutaMAX and sodium pyruvate + 10% heat‐inactivated FBS. 24 and 48 h later, viral supernatant was collected, filtered with a 0.45 µm PVDF filter (CELLTREAT) and pooled for cell transduction or stored short‐term (<1 week) at 4°C or long‐term at −80°C.

### 4.4 Dimeric GFP (dGFP) Production and Purification

*dGFP Cloning*: A mutated superfolder GFP sequence was used as the basis of the GFP dimer for copper‐mediated disulfide‐based dimerization, based on a previously reported amino acid sequence [43]. Two naturally occurring cysteines, C48 and C70, were mutated to alanine to reduce off‐target disulfide bonds, while the aspartic acid residue, D117, was mutated to cysteine for disulfide bonding. In addition, a His‐tag was added for protein purification. The mutated superfolder GFP coding sequence was produced through Thermo Fisher Scientific's GeneArt service and cloned into a pBH4 backbone for protein production.

### 4.5 Protein Expression

Plasmids containing the mutant GFP dimer sequence were transformed into BL21‐DE3 *E. coli* competent cells (NEB) and plated on an LB+agar with ampicillin supplementation plate for overnight incubation at 37°C. Colonies were then picked for three 4 mL starter cultures to be grown with ampicillin supplementation overnight at 37°C. The next day, the starter cultures were added into 1L of LB broth supplemented with ampicillin and allowed to grow at 37°C until the OD was measured in the 0.6–0.8 range. The culture was then induced with 1 mM IPTG (Gold Bio) and incubated for another 4 h at 37°C. Then the culture was spun down at 4000x *g* for 20 min to harvest the cells. The pellets were then consolidated into 5 mL of PBS and then spun down again at 4000x *g* for 10 min. Pellets were frozen and stored at −20°C for up to 2 months before purification. Cell pellets were lysed using the BugBuster (Novagen) reagent. Briefly, the wet weight of the pellet was determined, and 5 mL of BugBuster reagent was added per 1.5 g of pellet. 10 µL of Lysonase reagent was then added to the suspension, which was then incubated at room temperature for 30 min on a rotating mixer. To remove insoluble cell debris, the suspension was then centrifuged at 4000x *g* for 1 h at 4°C.

### 4.6 Protein Purification

His‐tagged protein was isolated using a cobalt resin column. After the suspension was passed through the gravity column, the column was washed with one column volume wash buffer (20 mM Tris‐HCl, 150 mM NaCl, 10 mM imidazole). The protein was then collected in fractions using elution buffer (20 mM Tris‐HCl, 150 mM NaCl, 250 mM imidazole). Fractions were pooled and then concentrated into 1 mL buffer (20 mM Tris pH 9.0, 100 mM NaCl) using Amicon molecular weight cutoff (MWCO) filters (Sigma‐Aldrich).

### 4.7 Dimerization

The GFP protein was then induced to form disulfide bonds using 10 mL dimerization buffer (20 mM Tris pH 9.0, 100 mM NaCl, 5 mM CuSO4). The reaction was allowed to proceed for 15 min at room temperature until quenching by addition of 50 mM EDTA. The protein was then exchanged into anion exchange buffer (10 mM Tris pH 9.5, 1 mM EDTA) using Amicon MWCO filters. Using an anion exchange column (Cytiva), the dimers were purified from the monomers via a salt gradient of 0–1 M NaCl in anion exchange buffer. Fractions were collected and validated using non‐reducing SDS‐PAGE. Fractions containing GFP dimers were desalted into DPBS using Amicon MWCO filters and aliquoted and frozen at −80°C.

### 4.8 Murine Mesenchymal Stem Cells

Bone‐marrow derived C57BL/6 mMSCs (Cyagen) were cultured in µEM‐α + GlutaMAX (Gibco) supplemented with 15% FBS in an incubator at 37°C and 5% carbon dioxide. For routine subcultivation, cells were rinsed with 1X DPBS (Gibco) and treated with TrypLE for 5 min at 37°C to detach cells. After quenching cells with complete medium, they were spun at 300x *g* for 5 min and replated in culture medium.

### 4.9 L929 Fibroblasts

Mouse L929 fibroblasts (ATCC# CCL‐1) were cultured in DMEM + GlutaMAX (Gibco) supplemented with 10% heat‐inactivated FBS in an incubator at 37°C and 5% carbon dioxide. For routine subcultivation, cells were treated with TrypLE for 5 min at 37°C to detach cells. After quenching cells with complete medium, they were spun at 300x *g* for 5 min and replated in culture medium.

### 4.10 RAW 264.7 Macrophages

RAW 264.7 macrophages (ATCC# TIB‐71) were cultured in DMEM + GlutaMAX (Gibco) supplemented with 10% FBS in an incubator at 37°C and 5% carbon dioxide. For routine sub‐cultivation, cells were rinsed with 1X DPBS (Gibco) and treated with TrypLE for 5 min at 37°C to detach cells. After quenching cells with complete medium, they were spun at 300x *g* for 5 min and replated in culture medium.

### 4.11 ATDC5 Chondrocytes

ATDC5s (Sigma Aldrich) were cultured in DMEM:F12 + GlutaMAX (Gibco) supplemented with 5% FBS in an incubator at 37°C and 5% carbon dioxide. For routine sub‐cultivation, cells were rinsed with 1X DPBS (Gibco) and treated with TrypLE for 5 min at 37°C to detach cells. After quenching cells with complete medium, they were spun at 300x*g* for 5 min and replated in culture medium.

### 4.12 Fibroblast‐Like Synoviocytes

Primary fibroblast‐like synoviocytes used for these studies were harvested from a single, 8‐week‐old C57BL/6 mice according to the following protocol [115]. Isolated FLS cells were cultured on adsorbed rat tail collagen I (Gibco) in DMEM + GlutaMAX (Gibco) supplemented with 10% FBS in an incubator at 37°C and 5% carbon dioxide. For routine sub‐cultivation, cells were rinsed with 1X DPBS (Gibco) and treated with TrypLE for 5 min at 37°C to detach cells. After quenching cells with complete medium, they were spun at 300x *g* for 5 min and replated on rat tail collagen I in culture medium.

### 4.13 Lentiviral Transduction

All cell lines were produced using reverse transduction using virus concentrated with LentiX Concentrator (Takara). Viral media was supplemented with 4 µg/mL of polybrene to enhance transduction efficiency and incubated with cells overnight at 37°C before being replaced with fresh cell culture media. Cells were selected using 10 µg/mL of puromycin (Sigma) when applicable. Unless otherwise noted, all cells following transduction were sorted for synNotch and transgene expression via fluorescence activated cell sorting (FACS).

### 4.14 Material‐Free Activation of Cytokine Responsive synNotch Receptors

mMSCs were co‐engineered with synNotch receptors with scFvs that each bind to one of TNF‐α, TGF‐β1, IL‐1β or IL‐6, and a transgene cassette expressing a tetracycline response element (TRE) driven mCherry and luciferase output. 8000 cells were plated in a white‐walled 96‐well plate (Corning) and treated with their respective ligands: TNF‐α (25 ng/mL, StemCellTech), TGF‐β1 (50 ng/mL, StemCellTech), IL‐1β (10 ng/mL, MedChemExpress) or IL‐6 (10 ng/mL, MedChemExpress). After 48 h, CellTiter‐Fluor (Promega) was performed according to manufacturer's instructions and read on a Tecan Infinite M1000 Pro plate reader. Following this, a BrightGlo (Promega) assay measuring firefly luminescence as a measure of synNotch activity was performed according to manufacturer's protocol on a Tecan Infinite M1000 Pro plate reader. Normalization was performed by dividing luminescence values each well's respective CellTiter‐Fluor output.

### 4.15 GFP Dimer Activation Assay

synNotch L929 fibroblasts were plated at a density of 18 000 cells/well (56 250 cells/cm^2^) on a control surface of streptavidin only. The LaG16 (K_d_ of 0.7 nM) synNotch receptor was exposed to a range of concentrations from 0 to 100 nM of either recombinant GFP purified from *E. coli* via immobilized metal affinity chromatography or mutant purified GFP dimer (purification process described above). After 48 h, firefly luminescence was measured using the BrightGlo luminescence assay (Promega) on a Tecan Infinite M1000 Pro plate reader. Results are expressed as fold change relative to GFP‐free conditions.

### 4.16 Antibody Surface Functionalization

Recombinant protein A/G (ThermoFisher) was reconstituted according to manufacturer's recommendations. Aliquoted Protein A/G was further diluted to 10ug/mL in BupH Carbonate‐Bicarbonate buffer (ThermoFisher) and 100uL per well was added to tissue culture (TC) treated 96‐well plates (Corning). After incubating 1 h at room temperature (RT), the solution was aspirated and washed with 200 µL of 1X DPBS (Gibco). Gevokizumab (MedChemExpress) or Olokizumab (MedChemExpress) were diluted to 10 µg/mL and 100 µL were added to the Protein A/G coated wells and left to coat overnight at 4°C. The following day, wells were washed with 200 µL of 1X DPBS and used immediately.

### 4.17 Biomaterial‐Based Activation of synNotch

After functionalizing a plate with gevokizumab, 12 000 canakinumab synNotch engineered mMSCs were plated in each well and supplemented with media containing IL‐1β in a range of concentrations from 0–100 ng/mL. After 48 h, firefly luminescence was measured via BrightGlo (Promega) luminescence assay on a Tecan Infinite M1000 Pro plate reader. To assess IL‐6 synNotch activation, this same procedure was performed using an olokizumab functionalized surface and ziltivekimab engineered synNotch cells dosed with 0–100 ng/mL of IL‐6.

### 4.18 IL‐1β ELISA

96‐well TC plates were coated with Protein A/G and gevokizumab as previously mentioned. 12 000 of either wild‐type or canakinumab synNotch engineered cells were plated in each well and dosed with 10 ng/mL of IL‐1β. After 48 h of culture, conditioned media were spun at 1500x *g* for 15 min and were analyzed using an IL‐1β ELISA kit (R&DSystems DY201) according to the manufacturer's protocol.

### 4.19 IL‐6 ELISA

96‐well TC plates were coated with Protein A/G and olokizumab as previously mentioned. 12 000 of either wild‐type or ziltivekimab synNotch engineered cells were plated in each well and dosed with 10 ng/mL of IL‐6. After 48 h of culture, conditioned media was spun at 1500x *g* for 15 min and was analyzed using an IL‐6 ELISA kit (R&DSystems DY206) according to the manufacturer's protocol.

### 4.20 NF‐κB Transcriptional Activity Assay

NF‐κB reporter mMSCs were generated via lentiviral transduction of a plasmid encoding four repeats of the NF‐κB response elements followed by a minimal promoter sequence [116], allowing for activation of a downstream luciferase transgene when NF‐κB is active. To assess modulation of native NF‐κB signaling, 96‐well TC plates were treated with Protein A/G and gevokizumab as previously described and 12 000 of either wild‐type or canakinumab engineered reporter mMSCs were added to each well and supplemented with 0, 1, or 10 ng/mL of IL‐1β in 100 µL of media. After 24 h, conditioned media from these groups were added to NF‐κB reporter mMSCs, which were plated at a density of 8000 cells per well in a 96‐well, white‐walled TC treated plate the day prior. After 5 h of co‐culture, a BrightGlo (Promega) luminescence assay was performed on the NF‐κB reporter cells to quantify NF‐κB activity.

### 4.21 Repeated Cytokine Stimulation of Canak‐Notch Cells

Gevokizumab‐functionalization was prepared as previously described. At *t* = 0, 2000 Canak‐Notch MSCs were plated onto the surface and dosed with 10 ng/mL IL‐1β. At *t* = 48 h, media were removed and cells were allowed to recover with IL‐1β‐free media for 24 h. This process was repeated two additional times, with IL‐1β being supplemented at *t* = 72 h and *t* = 144 h and incubated for 48 h each time. To measure synNotch activity, conditioned media were removed from cells and 100 µL of 150 µM D‐Luciferin potassium salt solution (Promega) was gently added to Canak‐Notch MSCs, and resultant firefly luminescence was measured via plate reader. Measurements were taken at *t* = 48, 72, 120, 144, and 196 h. Simultaneously, conditioned media from Canak‐Notch MSCs were centrifuged at 1500x *g* for 5 min in a U‐bottom plate to remove any cells, and conditioned media were added to 8000 NF‐κB reporter MSCs plated 24 h prior. Resultant luminescence was measured 5 h later via BrightGlo (Promega) luminescence assay to quantify NF‐κB activity. Three total measurements were taken at *t* = 48, 120, and 196 h.

### 4.22 SynNotch and SNIPR Activation

IL‐1β and IL‐6 SNIPR receptors (Canak‐SNIPR and Zilti‐SNIPR, respectively) were created by replacing the transmembrane domain of their synNotch counterparts with the CD8 Hinge, Notch 1TM and Notch2JM domains derived from an anti‐CD19 SNIPR construct (Addgene #188375) [34] via PCR and Gibson assembly. A flexible (G3S)x3 linker was added between the scFv and CD8 Hinge domains for additional flexibility. 12,000 mMSCs expressing either canakinumab engineered synNotch or canakinumab engineered SNIPR and a reporter luciferase transgene were plated on a white‐walled, 96‐well plate. Wells were treated with gevokizumab as previously described. Cells were treated with 10 ng/mL of IL‐1β and a BrightGlo luminescence assay was performed 48 h after plating to assess receptor activity. The same procedure was performed to assess IL‐6 mediated activity of ziltivekimab engineered synNotch and SNIPR cells plated on olokizumab functionalized surfaces.

### 4.23 GFP‐Mediated Macrophage Modulation

Anti‐GFP LaG16‐synNotch engineered cells were transduced with a synNotch inducible transgene circuit that expresses mouse IL‐4 and mouse IL‐10 in response to activation. Wells of a 24 well non‐TC plate were coated with GFP‐TRAP‐PEG as previously described [38] and 80 000 mMSCs were plated and treated with 5 nM GFP. In a separate TC‐treated 24‐well plate, 180,000 RAW264.7 macrophages were plated. After 24 h, conditioned media from the anti‐GFP mMSCs were spun down at 1500x *g* for 10 min and added to the RAW264.7 cells. Media without GFP were added to the anti‐GFP mMSCs and were allowed to incubate another 24 h before being transferred to the RAW264.7 macrophages as previously described. After an additional 24 h of incubation, RAW264.7 were lifted using Accutase and labeled for CD86, CD163, and CD206 surface markers using antibodies listed in Table S3 before undergoing flow cytometry.

### 4.24 IL‐1β‐Mediated Macrophage Modulation

Canakinumab‐synNotch mMSCs were engineered with the mouse IL‐4/IL‐10 payload via lentiviral transduction as described earlier. 80,000 mMSCs were added to the basal chamber of a gevokizumab functionalized Transwell plate (Corning) and were treated with 10 ng/mL of IL‐1β. Simultaneously, 30 000 RAW264.7 macrophages were added to the apical chamber of a Transwell and kept in a separate well with 500 µL of media. After 24 h of incubation, the RAW264.7 Transwell inserts were transferred into the mMSC wells and allowed to incubate for an additional 48 h. After co‐culturing, RAW264.7 cells were lifted using Accutase and stained for CD86, CD163 and CD206 surface markers before undergoing flow cytometry.

### 4.25 mIL‐4 and mIL‐10 ELISA

Mouse IL‐4 and mouse IL‐10 ELISA levels were assessed using respective ELISA kits (R&DSystems, DY404 and DY417) according to manufacturer instructions.

### 4.26 Flow Cytometry

All quantitative flow cytometry was performed on Amnis Cellstream flow cytometer. Cells were resuspended in DPBS supplemented with 2% FBS. Fluorescence activated cell sorting (FACS) was performed at the Vanderbilt Flow Core using a BD 5 Laser FACS Aria. Antibodies were diluted according to manufacturer's instructions for all samples. A list of antibodies used can be found in Table S2.

### 4.27 Statistical Analysis

Graphs were produced using GraphPad Prism 10. All bar graphs display the mean of triplicate values with errors bars showing the standard deviation. To determine significance in experiments, a two‐ or three‐way ANOVA with an alpha of 0.05 was used to compare between groups as needed. ANOVA tests were conducted along with Tukey's post‐hoc test with multiple comparisons.

## Author Contributions

**Zachary M. Eidman**: conceptualization, methodology, validation, writing – original draft, visualization, formal analysis, investigation, writing – review and editing, funding acquisition. **Joanne C. Lee**: validation, formal analysis, investigation, writing – original draft, writing – review and editing. **Marjan Rafat**: resources, writing – review and editing. **Nicholas F. Schulze**: validation, investigation, formal analysis, writing – original draft, writing – review and editing. **Jhanvi Sharma**: conceptualization, investigation, writing – original draft, methodology, validation, writing – review and editing, formal analysis. **Kevin C. Corn**: resources, writing – review and editing. **Hannah J. Brien**: validation, formal analysis, investigation, writing – original draft, writing – review and editing, visualization. **Jonathan M. Brunger**: conceptualization, formal analysis, validation, supervision, resources, project administration, writing – original draft, writing – review and editing, funding acquisition, methodology.

## Conflicts of Interest

JMB has filed patent applications pertaining to technology presented here.

## Supporting information




**Supporting File**: adhm71481‐sup‐0001‐SuppMat.pdf.

## Data Availability

The data that support the findings of this study are available from the corresponding author upon reasonable request.

## References

[1] Z. M. Jessop , A. Al‐Sabah , W. R. Francis , and I. S. Whitaker , “Transforming Healthcare Through Regenerative Medicine,” BMC Medicine 14 (2016): 115, 10.1186/s12916-016-0669-4. 27510095 PMC4980802

[2] D. Goldenberg , C. McLaughlin , S. V. Koduru , and D. J. Ravnic , “Regenerative Engineering: Current Applications and Future Perspectives,” Frontiers in Surgery 8 (2021): 731031, 10.3389/fsurg.2021.731031. 34805257 PMC8595140

[3] C. J. Esdaille , K. S. Washington , and C. T. Laurencin , “Regenerative Engineering: A Review of Recent Advances and Future Directions,” Regenerative Medicine 16 (2021): 495–512, 10.2217/rme-2021-0016. 34030463 PMC8356698

[4] D. Salthouse , K. Novakovic , C. M. U. Hilkens , and A. M. Ferreira , “Interplay Between Biomaterials and the Immune System: Challenges and Opportunities in Regenerative Medicine,” Acta Biomaterialia 155 (2023): 1–18, 10.1016/j.actbio.2022.11.003. 36356914

[5] C. M. Weyand and J. J. Goronzy , “The Immunology of Rheumatoid Arthritis,” Nature Immunology 22 (2021): 10–18, 10.1038/s41590-020-00816-x. 33257900 PMC8557973

[6] V. Turinetto , E. Vitale , and C. Giachino , “Senescence in Human Mesenchymal Stem Cells: Functional Changes and Implications in Stem Cell‐Based Therapy,” International Journal of Molecular Sciences 17 (2016): 1164, 10.3390/ijms17071164. 27447618 PMC4964536

[7] C. N. Serhan , “Treating Inflammation and Infection in the 21st Century: New Hints From Decoding Resolution Mediators and Mechanisms,” FASEB Journal 31 (2017): 1273–1288, 10.1096/fj.201601222R. 28087575 PMC5349794

[8] Y. Li , Y. Xiao , and C. Liu , “The Horizon of Materiobiology: A Perspective on Material‐Guided Cell Behaviors and Tissue Engineering,” Chemical Reviews 117 (2017): 4376–4421, 10.1021/acs.chemrev.6b00654. 28221776

[9] Z. Tu , Y. Zhong , H. Hu , et al., “Design of Therapeutic Biomaterials to Control Inflammation,” Nature Reviews Materials 7 (2022): 557–574, 10.1038/s41578-022-00426-z. 35251702 PMC8884103

[10] R. B. Kargbo , “Advances in Targeted Therapies for Inflammatory Diseases and Cancer: Exploring Cellular Mechanisms and Therapeutic Strategies,” ACS Medicinal Chemistry Letters 15 (2024): 758–760, 10.1021/acsmedchemlett.4c00182. 38894902 PMC11181491

[11] A. M. Rosales and K. S. Anseth , “The Design of Reversible Hydrogels to Capture Extracellular Matrix Dynamics,” Nature Reviews Materials 1 (2016): 15012, 10.1038/natrevmats.2015.12. PMC5714327 29214058

[12] C. M. Kirschner and K. S. Anseth , “Hydrogels in Healthcare: From Static to Dynamic Material Microenvironments,” Acta Materialia 61 (2013): 931–944, 10.1016/j.actamat.2012.10.037. 23929381 PMC3735227

[13] M. Guvendiren and J. A. Burdick , “Stiffening Hydrogels to Probe Short‐ and Long‐Term Cellular Responses to Dynamic Mechanics,” Nature Communications 3 (2012): 792, 10.1038/ncomms1792. 22531177

[14] J. R. Martin , M. K. Gupta , J. M. Page , et al., “A Porous Tissue Engineering Scaffold Selectively Degraded by Cell‐Generated Reactive Oxygen Species,” Biomaterials 35 (2014): 3766–3776, 10.1016/j.biomaterials.2014.01.026. 24491510 PMC3975079

[15] J. R. Martin , P. Patil , F. Yu , M. K. Gupta , and C. L. Duvall , “Enhanced Stem Cell Retention and Antioxidative Protection With Injectable, ROS‐Degradable PEG Hydrogels,” Biomaterials 263 (2020): 120377, 10.1016/j.biomaterials.2020.120377. 32947094 PMC8112229

[16] Y. Wu , Y. Wang , L. Long , C. Hu , Q. Kong , and Y. Wang , “A Spatiotemporal Release Platform Based on pH/ROS Stimuli‐Responsive Hydrogel in Wound Repairing,” Journal of Controlled Release 341 (2022): 147–165, 10.1016/j.jconrel.2021.11.027. 34813880

[17] M. G. Bezold , A. R. Hanna , B. R. Dollinger , et al., “Hybrid Shear‐Thinning Hydrogel Integrating Hyaluronic Acid With ROS‐Responsive Nanoparticles,” Advanced Functional Materials 33 (2023): 2213368, 10.1002/adfm.202213368. 38107427 PMC10723243

[18] E. R. Ruskowitz , M. P. Comerford , B. A. Badeau , and C. A. DeForest , “Logical Stimuli‐Triggered Delivery of Small Molecules From Hydrogel Biomaterials,” Biomaterials Science 7 (2019): 542–546, 10.1039/C8BM01304G. 30556545

[19] J. L. Holloway , H. Ma , R. Rai , K. D. Hankenson , and J. A. Burdick , “Synergistic Effects of SDF‐1α and BMP‐2 Delivery From Proteolytically Degradable Hyaluronic Acid Hydrogels for Bone Repair,” Macromolecular Bioscience 15 (2015): 1218–1223, 10.1002/mabi.201500178. 26059079 PMC4558375

[20] D. E. Meyer , B. C. Shin , G. A. Kong , M. W. Dewhirst , and A. Chilkoti , “Drug Targeting Using Thermally Responsive Polymers and Local Hyperthermia,” Journal of Controlled Release 74 (2001): 213–224, 10.1016/S0168-3659(01)00319-4. 11489497

[21] Y. Zhang , R. Wang , Y. Hua , R. Baumgartner , and J. Cheng , “Trigger‐Responsive Poly(β‐amino ester) Hydrogels,” ACS Macro Letters 3 (2014): 693–697, 10.1021/mz500277j. 35590770

[22] B. P. Purcell , D. Lobb , M. B. Charati , et al., “Injectable and Bioresponsive Hydrogels for On‐Demand Matrix Metalloproteinase Inhibition,” Nature Materials 13 (2014): 653–661, 10.1038/nmat3922. 24681647 PMC4031269

[23] I. Batalov , K. R. Stevens , and C. A. DeForest , “Photopatterned Biomolecule Immobilization to Guide Three‐Dimensional Cell Fate in Natural Protein‐Based Hydrogels,” Proceedings of the National Academy of Sciences 118 (2021): e2014194118, 10.1073/pnas.2014194118. PMC7848611 33468675

[24] M. L. Ohnsorg , E. A. Hushka , and K. S. Anseth , “Photoresponsive Chemistries for User‐Directed Hydrogel Network Modulation to Investigate Cell–Matrix Interactions,” Accounts of Chemical Research 58 (2025): 47–60, 10.1021/acs.accounts.4c00548. 39665396 PMC12950295

[25] A. M. Kloxin , M. W. Tibbitt , and K. S. Anseth , “Synthesis of Photodegradable Hydrogels as Dynamically Tunable Cell Culture Platforms,” Nature Protocols 5 (2010): 1867–1887, 10.1038/nprot.2010.139. 21127482 PMC3897936

[26] H. Wang , R. T. T. Morales , X. Cui , et al., “A Photoresponsive Hyaluronan Hydrogel Nanocomposite for Dynamic Macrophage Immunomodulation,” Advanced Healthcare Materials 8 (2019): 1801234, 10.1002/adhm.201801234. PMC6392032 30537061

[27] T. A. Baeumler , A. A. Ahmed , and T. A. Fulga , “Engineering Synthetic Signaling Pathways With Programmable dCas9‐Based Chimeric Receptors,” Cell Reports 20 (2017): 2639–2653, 10.1016/j.celrep.2017.08.044. 28903044 PMC5608971

[28] L. Morsut , K. T. Roybal , X. Xiong , et al., “Engineering Customized Cell Sensing and Response Behaviors Using Synthetic Notch Receptors,” Cell 164 (2016): 780–791, 10.1016/j.cell.2016.01.012. 26830878 PMC4752866

[29] N. M. Daringer , R. M. Dudek , K. A. Schwarz , and J. N. Leonard , “Modular Extracellular Sensor Architecture for Engineering Mammalian Cell‐Based Devices,” ACS Synthetic Biology 3 (2014): 892–902, 10.1021/sb400128g. 24611683 PMC4161666

[30] N. H. Kipniss , P. Dingal , T. R. Abbott , et al., “Engineering Cell Sensing and Responses Using a GPCR‐Coupled CRISPR‐Cas System,” Nature Communications 8 (2017): 2212, 10.1038/s41467-017-02075-1. PMC5738360 29263378

[31] K. Krawczyk , L. Scheller , H. Kim , and M. Fussenegger , “Rewiring of Endogenous Signaling Pathways to Genomic Targets for Therapeutic Cell Reprogramming,” Nature Communications 11 (2020): 608, 10.1038/s41467-020-14397-8. PMC6992713 32001704

[32] L. Scheller , T. Strittmatter , D. Fuchs , D. Bojar , and M. Fussenegger , “Generalized Extracellular Molecule Sensor Platform for Programming Cellular Behavior,” Nature Chemical Biology 14 (2018): 723–729, 10.1038/s41589-018-0046-z. 29686358

[33] D. I. Piraner , M. H. Abedi , M. J. Duran Gonzalez , et al., “Engineered Receptors for Soluble Cellular Communication and Disease Sensing,” Nature 638 (2025): 805–813, 10.1038/s41586-024-08366-0. 39542025 PMC11839477

[34] I. Zhu , R. Liu , J. M. Garcia , et al., “Modular Design of Synthetic Receptors for Programmed Gene Regulation in Cell Therapies,” Cell 185 (2022): 1431–1443.e16, 10.1016/j.cell.2022.03.023. 35427499 PMC9108009

[35] G. Barnea , W. Strapps , G. Herrada , et al., “The Genetic Design of Signaling Cascades to Record Receptor Activation,” Proceedings of the National Academy of Sciences 105 (2008): 64–69, 10.1073/pnas.0710487105. PMC2224232 18165312

[36] D. Lee , M. Creed , K. Jung , et al., “Temporally Precise Labeling and Control of Neuromodulatory Circuits in the Mammalian Brain,” Nature Methods 14 (2017): 495–503, 10.1038/nmeth.4234. 28369042

[37] N. A. Kalogriopoulos , R. Tei , Y. Yan , et al., “Synthetic GPCRs for Programmable Sensing and Control of Cell Behaviour,” Nature 637 (2025): 230–239, 10.1038/s41586-024-08282-3. 39633047 PMC11666456

[38] J. C. Lee , H. J. Brien , B. L. Walton , et al., “Instructional Materials That Control Cellular Activity Through Synthetic Notch Receptors,” Biomaterials 297 (2023): 122099, 10.1016/j.biomaterials.2023.122099. 37023529 PMC10320837

[39] B. Varnum‐Finney , L. Wu , M. Yu , et al., “Immobilization of Notch Ligand, Delta‐1, Is Required for Induction of Notch Signaling,” Journal of Cell Science 113 (2000): 4313–4318, 10.1242/jcs.113.23.4313. 11069775

[40] T. Huang , L. David , V. Mendoza , et al., “TGF‐β Signalling is Mediated by Two Autonomously Functioning TβRI:TβRII Pairs,” EMBO Journal 30 (2011): 1263–1276, 10.1038/emboj.2011.54. 21423151 PMC3094126

[41] R. A. Smith and C. Baglioni , “The Active Form of Tumor Necrosis Factor is a Trimer,” Journal of Biological Chemistry 262 (1987): 6951–6954, 10.1016/S0021-9258(18)48183-5. 3034874

[42] Y. A. Muller , B. Li , H. W. Christinger , J. A. Wells , B. C. Cunningham , and A. M. de Vos , “Vascular Endothelial Growth Factor: Crystal Structure and Functional Mapping of the Kinase Domain Receptor Binding Site,” Proceedings of the National Academy of Sciences 94 (1997): 7192–7197, 10.1073/pnas.94.14.7192. PMC23789 9207067

[43] D. J. Leibly , M. A. Arbing , I. Pashkov , et al., “A Suite of Engineered GFP Molecules for Oligomeric Scaffolding,” Structure 23 (2015): 1754–1768, 10.1016/j.str.2015.07.008. 26278175 PMC4568552

[44] P. Zhong , Z. Wang , S. Cheng , et al., “Structural Insights Into Two Distinct Nanobodies Recognizing the Same Epitope of Green Fluorescent Protein,” Biochemical and Biophysical Research Communications 565 (2021): 57–63, 10.1016/j.bbrc.2021.05.089. 34098312

[45] M. Blech , D. Peter , P. Fischer , et al., “One Target—Two Different Binding Modes: Structural Insights Into Gevokizumab and Canakinumab Interactions to Interleukin‐1β,” Journal of Molecular Biology 425 (2013): 94–111, 10.1016/j.jmb.2012.09.021. 23041424

[46] U. Bilitewski , “Protein‐Sensing Assay Formats and Devices,” Analytica Chimica Acta 568 (2006): 232–247, 10.1016/j.aca.2005.12.073. 17761265

[47] W.‐W. Lin , Y.‐C. Lu , B.‐C. Huang , et al., “Selective Activation of Pro‐Anti‐IL‐1β Antibody Enhances Specificity for Autoinflammatory Disorder Therapy,” Scientific Reports 11 (2021): 14846, 10.1038/s41598-021-94298-y. 34290297 PMC8295355

[48] S.‐Y. Park , M.‐J. Kang , and J.‐S. Han , “Interleukin‐1 Beta Promotes Neuronal Differentiation Through the Wnt5a/RhoA/JNK Pathway in Cortical Neural Precursor Cells,” Molecular Brain 11 (2018): 39, 10.1186/s13041-018-0383-6. 29973222 PMC6033214

[49] A. Cordeiro‐Santanach , F. Morales , M. D. C. Parquet , et al., “The Effect of IL‐1β Inhibitor canakinumab (Ilaris®) on IL‐6 Production in human Skeletal Muscle Cells,” PLoS ONE 20 (2025): 0316110, 10.1371/journal.pone.0316110. PMC11884680 40048444

[50] C. A. Nold‐Petry , M. F. Nold , J. A. Zepp , S.‐H. Kim , N. F. Voelkel , and C. A. Dinarello , “IL‐32–Dependent Effects of IL‐1β on Endothelial Cell Functions,” Proceedings of the National Academy of Sciences 106 (2009): 3883–3888, 10.1073/pnas.0813334106. PMC2656174 19228941

[51] H. Zhong , A. Davis , M. Ouzounova , et al., “A Novel IL6 Antibody Sensitizes Multiple Tumor Types to Chemotherapy Including Trastuzumab‐Resistant Tumors,” Cancer Research 76 (2016): 480–490, 10.1158/0008-5472.CAN-15-0883. 26744529

[52] S. Shaw , T. Bourne , C. Meier , et al., “Discovery and Characterization of Olokizumab,” MAbs 6 (2014): 773–781, 10.4161/mabs.28612. PMC4011921 24670876

[53] S. Kang , T. Tanaka , H. Inoue , et al., “IL‐6 Trans‐signaling Induces Plasminogen Activator Inhibitor‐1 From Vascular Endothelial Cells in Cytokine Release Syndrome,” Proceedings of the National Academy of Sciences 117 (2020): 22351–22356, 10.1073/pnas.2010229117. PMC7486751 32826331

[54] A. Latourte , C. Cherifi , J. Maillet , et al., “Systemic Inhibition of IL‐6/Stat3 Signalling Protects Against Experimental Osteoarthritis,” Annals of the Rheumatic Diseases 76 (2017): 748–755, 10.1136/annrheumdis-2016-209757. 27789465

[55] M. A. Huseni , L. Wang , J. E. Klementowicz , et al., “CD8+ T Cell‐Intrinsic IL‐6 Signaling Promotes Resistance to Anti‐PD‐L1 Immunotherapy,” Cell Reports Medicine 4 (2023): 100878, 10.1016/j.xcrm.2022.100878. 36599350 PMC9873827

[56] M. Otero and M. B. Goldring , “Cells of the Synovium in Rheumatoid Arthritis. Chondrocytes,” Arthritis Research & Therapy 9 (2007): 220, 10.1186/ar2292. 18001488 PMC2212563

[57] B. Bartok and G. S. Firestein , “Fibroblast‐Like Synoviocytes: Key Effector Cells in rheumatoid arthritis,” Immunological Reviews 233 (2010): 233–255, 10.1111/j.0105-2896.2009.00859.x. 20193003 PMC2913689

[58] A. J. Knights , E. C. Farrell , O. M. Ellis , M. J. Song , C. T. Appleton , and T. Maerz , “Synovial Macrophage Diversity and Activation of M‐CSF Signaling in Post‐Traumatic Osteoarthritis,” Elife 12: RP93283, 10.7554/eLife.93283. 39969512 PMC11839164

[59] A. J. Knights , S. J. Redding , and T. Maerz , “Inflammation in Osteoarthritis: The Latest Progress and Ongoing Challenges,” Current Opinion in Rheumatology 35 (2023): 128–134, 10.1097/BOR.0000000000000923. 36695054 PMC10821795

[60] C.‐L. Wu , N. S. Harasymowicz , M. A. Klimak , K. H. Collins , and F. Guilak , “The Role of Macrophages in Osteoarthritis and Cartilage Repair,” Osteoarthritis and Cartilage 28 (2020): 544–554, 10.1016/j.joca.2019.12.007. 31926267 PMC7214213

[61] R. Liao , Z. Feng , W. Li , et al., “Interleukin‑1 Induces Receptor Activator of Nuclear Factor‑κB ligand‑Independent Osteoclast Differentiation in RAW264.7 Cells,” Experimental and Therapeutic Medicine 21 (2021): 640, 10.3892/etm.2021.10072. 33968171 PMC8097200

[62] P. LI , C. MA , J. LI , et al., “Proteomic Characterization of Four Subtypes of M2 Macrophages Derived From Human THP‐1 Cells,” Journal of Zhejiang University‐SCIENCE B 23 (2022): 407–422, 10.1631/jzus.B2100930. 35557041 PMC9110321

[63] J. Hwang , M. Zheng , C. Wiraja , M. Cui , L. Yang , and C. Xu , “Reprogramming of Macrophages With Macrophage Cell Membrane‐Derived Nanoghosts,” Nanoscale Advances 2: 5254–5262, 10.1039/d0na00572j. PMC9419214 36132036

[64] B. Benner , L. Scarberry , L. P. Suarez‐Kelly , et al., “Generation of Monocyte‐DerivedTumor‐Associated Macrophages Using Tumor‐Conditioned Media Provides a Novel Method to Study Tumor‐Associated Macrophages In Vitro,” Journal for ImmunoTherapy of Cancer 7 (2019): 140, 10.1186/s40425-019-0622-0. 31138333 PMC6540573

[65] D. Fairweather and D. Cihakova , “Alternatively Activated Macrophages in Infection and Autoimmunity,” Journal of Autoimmunity 33 (2009): 222–230, 10.1016/j.jaut.2009.09.012. 19819674 PMC2783278

[66] F. O. Martinez , “Macrophage Activation and Polarization,” Frontiers in Bioscience 13 (2008): 453–461, 10.2741/2692. 17981560

[67] Y.‐X. Zeng , K.‐Y. Chou , J.‐J. Hwang , and H.‐S. Wang , “The Effects of IL‐1β Stimulated Human Umbilical Cord Mesenchymal Stem Cells on Polarization and Apoptosis of Macrophages in Rheumatoid Arthritis,” Scientific Reports 13 (2023): 10612, 10.1038/s41598-023-37741-6. 37391581 PMC10313744

[68] C. Yang , J. Dawulieti , K. Zhang , et al., “An Injectable Antibiotic Hydrogel That Scavenges Proinflammatory Factors for the Treatment of Severe Abdominal Trauma,” Advanced Functional Materials 32 (2022): 2111698, 10.1002/adfm.202111698.

[69] H. Shen , B. Xu , C. Yang , et al., “A DAMP‐scavenging, IL‐10‐releasing Hydrogel Promotes Neural Regeneration and Motor Function Recovery After Spinal Cord Injury,” Biomaterials 280 (2022): 121279, 10.1016/j.biomaterials.2021.121279. 34847433

[70] S. Kumar , T. Conklin , J. Hsu , et al., “Controlled Release of CD200 Inhibits Inflammatory Macrophages and Chondrocyte Catabolism,” Osteoarthritis and Cartilage 30 (2022): S69–S70, 10.1016/j.joca.2022.02.083. PMC13421671 42527473

[71] A. Y. Clark , K. E. Martin , J. R. García , et al., “Integrin‐Specific Hydrogels Modulate Transplanted Human Bone Marrow‐Derived Mesenchymal Stem Cell Survival, Engraftment, and Reparative Activities,” Nature Communications 11 (2020): 114, 10.1038/s41467-019-14000-9. PMC6949269 31913286

[72] S. Cao , E. Budina , R. Wang , et al., “Injectable Butyrate‐prodrug Micelles Induce Long‐Acting Immune Modulation and Prevent Autoimmune Arthritis in Mice,” Journal of Controlled Release 372 (2024): 281–294, 10.1016/j.jconrel.2024.06.027. 38876359

[73] L. C. Ionescu , G. C. Lee , B. J. Sennett , J. A. Burdick , and R. L. Mauck , “An Anisotropic Nanofiber/Microsphere Composite With Controlled Release of Biomolecules for Fibrous Tissue Engineering,” Biomaterials 31 (2010): 4113–4120, 10.1016/j.biomaterials.2010.01.098. 20149432 PMC2838985

[74] A. R. Martin , J. M. Patel , R. C. Locke , et al., “Nanofibrous Hyaluronic Acid Scaffolds Delivering TGF‐β3 and SDF‐1α for Articular Cartilage Repair in a Large Animal Model,” Acta Biomaterialia 126 (2021): 170–182, 10.1016/j.actbio.2021.03.013. 33753316 PMC8314069

[75] K. M. Hainline , L. S. Shores , N. L. Votaw , et al., “Modular Complement Assemblies for Mitigating Inflammatory Conditions,” Proceedings of the National Academy of Sciences 118 (2021): e2018627118, 10.1073/pnas.2018627118. PMC8054013 33876753

[76] S. K. Bedingfield , J. M. Colazo , F. Yu , et al., “Amelioration of Post‐Traumatic Osteoarthritis via Nanoparticle Depots Delivering Small Interfering RNA to Damaged Cartilage,” Nature Biomedical Engineering 5 (2021): 1069–1083, 10.1038/s41551-021-00780-3. PMC8497446 34413494

[77] X. Huang , J. Z. Williams , R. Chang , et al., “DNA Scaffolds Enable Efficient and Tunable Functionalization of Biomaterials for Immune Cell Modulation,” Nature Nanotechnology 16 (2021): 214–223, 10.1038/s41565-020-00813-z. PMC7878327 33318641

[78] H. Yin , Q. Lu , X. Wang , et al., “Tissue‐Derived Microparticles Reduce Inflammation and Fibrosis in Cornea Wounds,” Acta Biomaterialia 85 (2019): 192–202, 10.1016/j.actbio.2018.12.027. 30579044 PMC9924072

[79] S. Regmi , S. Pathak , J. O. Kim , C. S. Yong , and J. H. Jeong , “Mesenchymal Stem Cell Therapy for the Treatment of Inflammatory Diseases: Challenges, Opportunities, and Future Perspectives,” European Journal of Cell Biology 98 (2019): 151041, 10.1016/j.ejcb.2019.04.002. 31023504

[80] M. Lopez‐Santalla , J. A. Bueren , and M. I. Garin , “Mesenchymal Stem/Stromal Cell‐Based Therapy for the Treatment of Rheumatoid Arthritis: An Update on Preclinical Studies,” EBioMedicine 69 (2021): 103427, 10.1016/j.ebiom.2021.103427. 34161884 PMC8237294

[81] M. Z. Moghaddam , M. J. Mousavi , and S. Ghotloo , “Cell‐Based Therapies for the Treatment of Rheumatoid Arthritis,” Immunity, Inflammation and Disease 11 (2023): 1091, 10.1002/iid3.1091. PMC10664399 38018576

[82] S. Wang , B. Lei , E. Zhang , et al., “Targeted Therapy for Inflammatory Diseases With Mesenchymal Stem Cells and Their Derived Exosomes: From Basic to Clinics,” International Journal of Nanomedicine 17 (2022): 1757–1781, 10.2147/IJN.S355366. 35469174 PMC9034888

[83] P. D. Robbins and C. H. Evans , “Prospects for Treating Autoimmune and Inflammatory Diseases by Gene Therapy,” Gene Therapy 3 (1996): 187–189. 8646548

[84] G. J. Prud'homme , “Gene Therapy of Autoimmune Diseases With Vectors Encoding Regulatory Cytokines or Inflammatory Cytokine Inhibitors,” Journal of Gene Medicine 2 (2000) 222–232, 10.1002/1521-2254(200007/08)2:4<222::AID-JGM117>3.0.CO;2-P. 10953913

[85] D. Ewart , E. J. Peterson , and C. J. Steer , “A New Era of Genetic Engineering for Autoimmune and Inflammatory Diseases,” Seminars in Arthritis and Rheumatism 49 (2019): e1–e7, 10.1016/j.semarthrit.2019.05.004. 31146955

[86] G. C. Abhiraman , K. D. Householder , G. E. Rodriguez , et al., “Redirecting Immune Signaling With Cytokine Adaptors,” Nature Communications 16 (2025): 2432, 10.1038/s41467-025-57681-1. PMC11897282 40069219

[87] R. W. Kinne , R. Bräuer , B. Stuhlmüller , E. Palombo‐Kinne , and G.‐R. Burmester , “Macrophages in Rheumatoid Arthritis,” Arthritis Research 2 (2000): 189, 10.1186/ar86. 11094428 PMC130001

[88] V. M. T. Bart , R. J. Pickering , P. R. Taylor , and N. Ipseiz , “Macrophage Reprogramming for Therapy,” Immunology 163 (2021): 128–144, 10.1111/imm.13300. 33368269 PMC8114216

[89] H. He , S. Zhang , S. Tighe , J. Son , and S. C. G. Tseng , “Immobilized Heavy Chain‐Hyaluronic Acid Polarizes Lipopolysaccharide‐Activated Macrophages Toward M2 Phenotype,” Journal of Biological Chemistry 288 (2013): 25792–25803, 10.1074/jbc.M113.479584. 23878196 PMC3764786

[90] E. Saino , M. L. Focarete , C. Gualandi , et al., “Effect of Electrospun Fiber Diameter and Alignment on Macrophage Activation and Secretion of Proinflammatory Cytokines and Chemokines,” Biomacromolecules 12 (2011): 1900–1911, 10.1021/bm200248h. 21417396

[91] A. Kerschenmeyer , Ø. Arlov , V. Malheiro , et al., “Anti‐Oxidant and Immune‐Modulatory Properties of Sulfated Alginate Derivatives on Human Chondrocytes and Macrophages,” Biomaterials Science 5 (2017): 1756–1765, 10.1039/C7BM00341B. 28643827

[92] K. Nakamura , S. Yokohama , M. Yoneda , et al., “High, but Not Low, Molecular Weight Hyaluronan Prevents T‐cell‐Mediated Liver Injury by Reducing Proinflammatory Cytokines in Mice,” Journal of Gastroenterology 39 (2004): 346–354, 10.1007/s00535-003-1301-x. 15168246

[93] T.‐H. Tran , R. Rastogi , J. Shelke , and M. M. Amiji , “Modulation of Macrophage Functional Polarity Towards Anti‐Inflammatory Phenotype With Plasmid DNA Delivery in CD44 Targeting Hyaluronic Acid Nanoparticles,” Scientific Reports 5 (2015): 16632, 10.1038/srep16632. 26577684 PMC4649614

[94] K. L. Spiller , S. Nassiri , C. E. Witherel , et al., “Sequential Delivery of Immunomodulatory Cytokines to Facilitate the M1‐to‐M2 Transition of Macrophages and Enhance Vascularization of Bone Scaffolds,” Biomaterials 37 (2015): 194–207, 10.1016/j.biomaterials.2014.10.017. 25453950 PMC4312192

[95] D. Kwon , B. G. Cha , Y. Cho , et al., “Extra‐Large Pore Mesoporous Silica Nanoparticles for Directing In Vivo M2 Macrophage Polarization by Delivering IL‐4,” Nano Letters 17 (2017): 2747–2756, 10.1021/acs.nanolett.6b04130. 28422506

[96] S. Jain , T.‐H. Tran , and M. Amiji , “Macrophage Repolarization With Targeted Alginate Nanoparticles Containing IL‐10 Plasmid DNA for the Treatment of Experimental Arthritis,” Biomaterials 61 (2015): 162–177, 10.1016/j.biomaterials.2015.05.028. 26004232 PMC4464978

[97] T. Gao , F. Huang , W. Wang , Y. Xie , and B. Wang , “Interleukin‐10 Genetically Modified Clinical‐Grade Mesenchymal Stromal Cells Markedly Reinforced Functional Recovery After Spinal Cord Injury via Directing Alternative Activation of Macrophages,” Cellular & Molecular Biology Letters 27 (2022): 27, 10.1186/s11658-022-00325-9. 35300585 PMC8931978

[98] S. T. Peruzzaro , M. M. M. Andrews , A. Al‐Gharaibeh , et al., “Transplantation of Mesenchymal Stem Cells Genetically Engineered to Overexpress Interleukin‐10 Promotes Alternative Inflammatory Response in Rat Model of Traumatic Brain Injury,” Journal of Neuroinflammation 16 (2019): 2, 10.1186/s12974-018-1383-2. 30611291 PMC6320578

[99] J. Gao , Y. Liang , and L. Wang , “Shaping Polarization of Tumor‐Associated Macrophages in Cancer Immunotherapy,” Frontiers in Immunology 13 (2022): 888713, 10.3389/fimmu.2022.888713. 35844605 PMC9280632

[100] G. Gross , T. Waks , and Z. Eshhar , “Expression of Immunoglobulin‐T‐Cell Receptor Chimeric Molecules as Functional Receptors With Antibody‐Type Specificity,” Proceedings of the National Academy of Sciences 86 (1989): 10024–10028, 10.1073/pnas.86.24.10024. PMC298636 2513569

[101] D. L. Porter , B. L. Levine , M. Kalos , A. Bagg , and C. H. June , “Chimeric Antigen Receptor–Modified T Cells in Chronic Lymphoid Leukemia,” New England Journal of Medicine 365 (2011): 725–733, 10.1056/NEJMoa1103849. 21830940 PMC3387277

[102] N. Ahmed , V. S. Brawley , M. Hegde , et al., “Human Epidermal Growth Factor Receptor 2 (HER2) –Specific Chimeric Antigen Receptor–Modified T Cells for the Immunotherapy of HER2‐Positive Sarcoma,” Journal of Clinical Oncology 33 (2015): 1688–1696, 10.1200/JCO.2014.58.0225. 25800760 PMC4429176

[103] Z. L. Chang , M. H. Lorenzini , X. Chen , U. Tran , N. J. Bangayan , and Y. Y. Chen , “Rewiring T‐Cell Responses to Soluble Factors With Chimeric Antigen Receptors,” Nature Chemical Biology 14 (2018): 317–324, 10.1038/nchembio.2565. 29377003 PMC6035732

[104] A. M. Leen , S. Sukumaran , N. Watanabe , et al., “Reversal of Tumor Immune Inhibition Using a Chimeric Cytokine Receptor,” Molecular Therapy 22 (2014): 1211–1220, 10.1038/mt.2014.47. 24732709 PMC4048899

[105] E. Engelowski , A. Schneider , M. Franke , et al., “Synthetic Cytokine Receptors Transmit Biological Signals Using Artificial Ligands,” Nature Communications 9 (2018): 2034, 10.1038/s41467-018-04454-8. PMC5964073 29789554

[106] H. I. Edelstein , A. Cosio , M. L. Ezekiel , W. K. Corcoran , A. H. Morris , and J. N. Leonard , “Conversion of Natural Cytokine Receptors Into Orthogonal Synthetic Biosensors,” Nature Chemical Biology (2025): 1–12, 10.1038/s41589-025-01986-1. 40846994 PMC12380174

[107] A. L. Z. Lee , V. W. L. Ng , S. Gao , J. L. Hedrick , and Y. Y. Yang , “Injectable Biodegradable Hydrogels From Vitamin D‐Functionalized Polycarbonates for the Delivery of Avastin With Enhanced Therapeutic Efficiency Against Metastatic Colorectal Cancer,” Biomacromolecules 16 (2015): 465–475, 10.1021/bm5015206. 25543531

[108] M. Dai , P. Du , Y. Li , et al., “Peptide‐Based Hydrogel Co‐Assembled With Antibody‐Drug for Enhanced Retinal Cell Uptake and Attenuated Experimental Autoimmune Uveitis,” European Journal of Pharmaceutics and Biopharmaceutics 210 (2025): 114691, 10.1016/j.ejpb.2025.114691. 40054506

[109] B. L. Walton , R. Shattuck‐Brandt , C. A. Hamann , et al., “A Programmable Arthritis‐Specific Receptor for Guided Articular Cartilage Regenerative Medicine,” Osteoarthritis and Cartilage 33 (2025): 231–240, 10.1016/j.joca.2024.12.002. 39706287 PMC12019866

[110] H. J. Brien , J. C. Lee , J. Sharma , et al., “Templated Pluripotent Stem Cell Differentiation via Substratum‐Guided Artificial Signaling,” ACS Biomaterials Science & Engineering 10 (2024): 6465–6482, 10.1021/acsbiomaterials.4c00885. 39352143 PMC11480943

[111] C. A. Hamann , A. Kjar , H. Kim , et al., “Induced Neural Progenitor Specification From Human Pluripotent Stem Cells by a Refined Synthetic Notch Platform,” ACS Synthetic Biology 14 (2025): 1482–1495, 10.1021/acssynbio.4c00742. 40327355 PMC12090341

[112] P. C. Fridy , Y. Li , S. Keegan , et al., “A Robust Pipeline for Rapid Production of Versatile Nanobody Repertoires,” Nature Methods 11 (2014): 1253–1260, 10.1038/nmeth.3170. 25362362 PMC4272012

[113] R. Zufferey , D. Nagy , R. J. Mandel , L. Naldini , and D. Trono , “Multiply Attenuated Lentiviral Vector Achieves Efficient Gene Delivery In Vivo,” Nature Biotechnology 15 (1997): 871–875, 10.1038/nbt0997-871. 9306402

[114] P. Salmon and D. Trono , “Production and Titration of Lentiviral Vectors,” Current Protocols in Human Genetics 54 (2007): 12.10.1‐12.10.24, 10.1002/0471142905.hg1210s54. 18428406

[115] M. Armaka , V. Gkretsi , D. Kontoyiannis , and G. Kollias , (2009), A standardized protocol for the isolation and culture of normal and arthritogenic murine synovial fibroblasts. 10.1038/nprot.2009.102.

[116] S. D. Perli , C. H. Cui , and T. K. Lu , “Continuous Genetic Recording With Self‐Targeting CRISPR‐Cas in Human Cells,” Science 353 (2016): aag0511, 10.1126/science.aag0511. 27540006

[117] 26

