# Supplementary material for: Encoded Cell‐Material Interactions to Reroute Cytokine Signaling for Regenerative Medicine
Source: Adv Healthc Mater. 2026 Jul 28;15(32):e71481. doi: 10.1002/adhm.71481 (PMC13507575; doi:10.1002/adhm.71481)
Supplement: Supplementary file 1 [file ADHM-15-0-s001.pdf]

Supplemental Materials and Methods:

Material-free activation of VEGF-responsive synNotch receptors: L929 fibroblasts were co-engineered with synNotch receptors with a VEGF binding scFv (Supplemental Table 1) and a transgene cassette expressing a tetracycline response element (TRE) driven luciferase output. 9,000 cells were plated in a white-walled 96-well plate (Corning) and treated with 25 ng/ml of VEGF (StemCellTech). After 48 hours, CellTiter-Fluor (Promega) was performed according to manufacturer's instructions and read on a Tecan Infinite M1000 Pro plate reader. Following this, a BrightGlo (Promega) assay measuring firefly luminescence as a measure of synNotch activity was performed according to manufacturer's protocol on a Tecan Infinite M1000 Pro plate reader. Normalization was performed by dividing luminescence values each well's respective CellTiter-Fluor output.

Maintenance of KOLF2.1J induced pluripotent stem cells: KOLF2.1J[1] stem cells were maintained in StemFlex (Gibco) in Geltrex (Gibco)-coated wells. Routine cell passages were carried out using ReLeSR (Stem Cell Technologies) to detach cells. For experiments, cells were dissociated into a single-cell suspension using Accutase (Gibco).

Establishing synNotch-KOLF2.1J iPSC line: KOLF2.1J iPSCs were engineered to stably express synNotch platform elements (i.e., receptor and transgene payloads) via a Sleeping Beauty transposase/transposon system. Using the TransIT®-LT1 Transfection Reagent (Mirus, #MIR 2300), 1 µg of the Sleeping Beauty 100x transposase (Addgene 34879, a kind gift from Zsuzsanna Izsvak) 46 and 1 µg of the transposon containing the synNotch receptor and transgene payloads were transfected into the iPSCs. Approximately 1 x 10^6 cells / mL were plated into a Geltrex-coated well in a 6-well plate and allowed to incubate with the DNA complexes for 24 hours at 37°C in StemFlex medium supplemented with 10 µM Y-27632 ROCK inhibitor (Tocris, #1254). After 24 hours, medium was replaced with fresh media. Engineered cells were selected with 0.6 µg/mL puromycin during sub-cultivation and sorted at the Vanderbilt Flow Cytometry Shared Resource with a 4-laser FACSAria III based on receptor expression using a c-myc-tag epitope appended to the synNotch receptor.

Activation of synNotch engineered iPSCs: To activate the GFP-responsive, LaG16 synNotch iPSCs, we supplemented StemFlex media with 0, 1, 5, 10, 50, 100, 500, or 1000 nM of dimeric sfGFP. Dimeric sfGFP was supplemented in the media when cells were plated and mCherry expression was assessed after 48 hours. To quantify mCherry expression, cells were fixed with 4% paraformaldehyde in DPBS for 10 minutes, blocked and (5% FBS and 0.3% Triton X in DPBS), and incubated with mCherry primary antibody (Supplemental Table 2) overnight at 4ºC. Cells were then washed three times with DPBS before incubation with an Alexa Fluor 647-conjugated Donkey anti-rabbit IgG secondary antibody (Supplemental Table 2) and the nuclear stain DAPI (Thermo Scientific, 62247, 1:1000). Cells were protected from light and incubated for 1 hour at room temperature. Cells were then washed with DPBS three times prior to imaging cells on a Leica Dmi8 microscope.

ImageJ analysis: Mean mCherry pixel intensity was calculated using ImageJ/Fiji software (available from the NIH at https://imagej.net/software/fiji/downloads) using built-in functions. Briefly, fluorescent images were converted to 8-bit greyscale images. DAPI channel images were used to develop a region of interest (ROI) that was used to measure the mean grey value in the mCherry channel. The ROI was made by subtracting out background noise (rolling ball radius = 50) and thresholding the greyscale image (85,255) to make a binary mask. Nuclei were then defined using the watershed feature prior to using the default analyze particles function to add the DAPI mask to the ROI manager. The ROI was then overlayed on the greyscale mCherry images to calculate the mean grey value for each nucleus. These values were then averaged to obtain the mean mCherry pixel intensity per nucleus.

Supplementary Table 1. Antibody variable fragments used to produce synNotch receptors.

scFv	Target	K_d
TNF3E [2]	TNF	16 pM
PET1074B9 [3]	TGF-β1	Not reported
G6-31 [4,5]	VEGF	0.9 nM
Canakinumab [6]	IL-1β	4.1 nM
Gevokizumab [6]	IL-1β	0.29 nM
Ziltivekimab [7]	IL-6	≤1 pM
Olokizumab [8]	IL-6	10 pM

Supplementary Table 2. Receptor construct names and Addgene repository information.

Plasmid name	Plasmid description	Addgene #
pLV EF1A LaG16-synNotch-tTA PGK puroR	Expresses GFP-sensitive synNotch receptor and puro antibiotic resistance	259284
pLV EF1A Canak-synNotch-tTA PGK puroR	Expresses IL-1β-sensitive synNotch receptor and puro antibiotic resistance	259285
pLV EF1A Zilti-synNotch-tTA PGK puroR	Expresses IL-6-sensitive synNotch receptor and puro antibiotic resistance	259287
pLV EF1A TNF3E-synNotch-tTA PGK puroR	Expresses TNF-sensitive synNotch receptor and puro antibiotic resistance	259288
pLV EF1A VEGF-synNotch-tTA PGK puroR	Expresses VEGF-sensitive synNotch receptor and puro antibiotic resistance	259289
pLV EF1A TGFB1-synNotch-tTA PGK puroR	Expresses TGFβ-1-sensitive synNotch receptor and puro antibiotic resistance	259290
pLV TRE mIL10-P2A-mIL4 PGK tagBFP	Inducible expression of murine IL-10 and IL-4	259291
pET-6xHis-TEV-dimeric sfGFP	IPTG-inducible expression of dimeric mutant form of superfolder GFP in bacteria	259292
pLV EF1A Canak-SNIPR-tTA PGK puroR	Expresses IL-1βsensitive synNotch receptor and puro antibiotic resistance	259293
pLV EF1A Zilti-synNotch-tTA PGK puroR	Expresses IL-6-sensitive synNotch receptor and puro antibiotic resistance	259294

Supplementary Table 3. Antibodies used for immunolabeling.

Antibody Target-label	Vendor	Dilution	Catalog #
Myc-Alexa Fluor 647	Cell Signaling Technology	1:50	2233S
Mouse CD86-PE	Biolegend	1:800	159203
Mouse CD163-Brilliant Violet 421	Biolegend	1:400	155309
Mouse CD206-Pacific Blue	Biolegend	1:400	141707
mCherry-Rabbit IgG	Cell Signaling Technology	1:200	43590
Donkey anti-Rabbit IgG- Alexa Fluor™ 647	Thermo Fisher Scientific	1:500	A31573

Supplementary Figures:

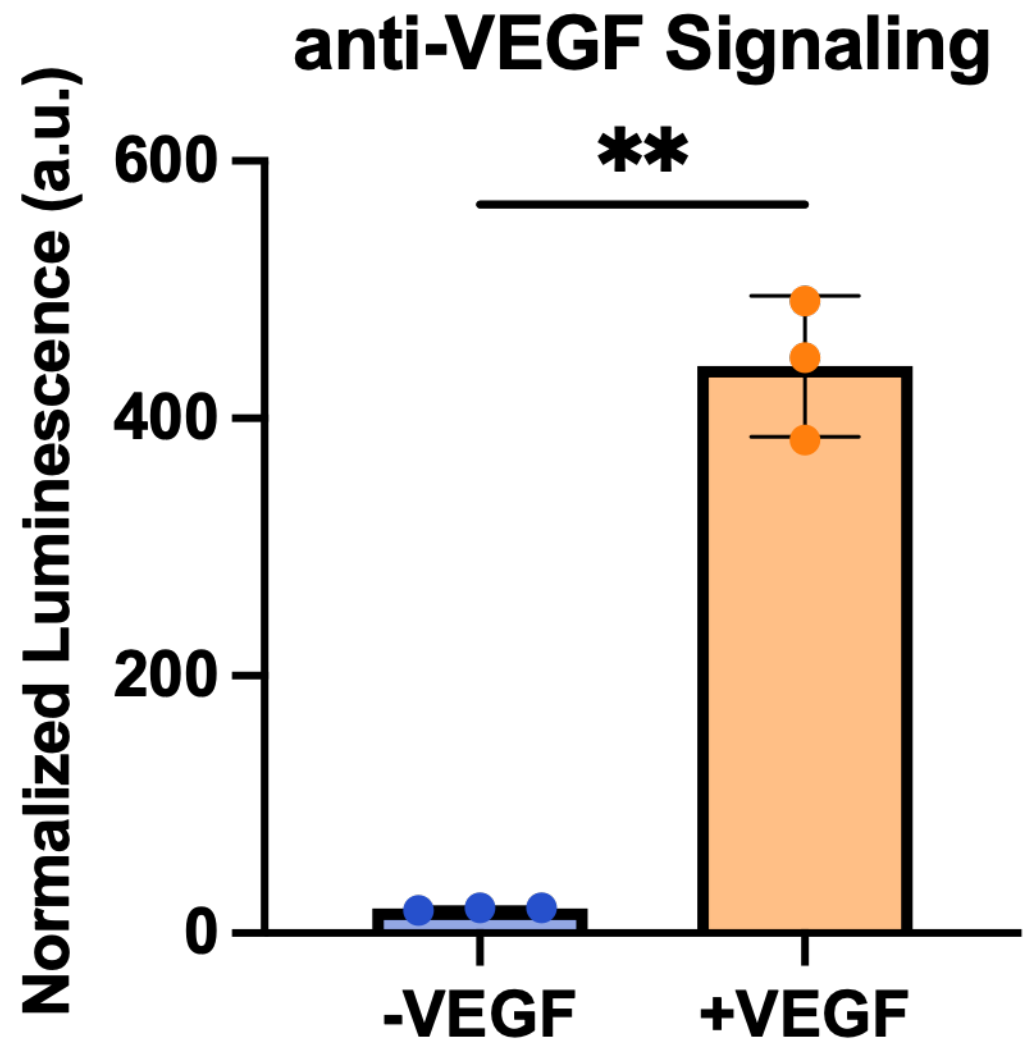

Supplementary Figure 1. Material independent VEGF synNotch signaling. L929 fibroblasts engineered with anti-VEGF synNotch demonstrate ligand dependent activation when supplemented with 25ng/mL of VEGF. Firefly luminescence readout normalized to cell viability. A Welch's t test of significance was performed. n=3 for all experimental groups **p<0.01.

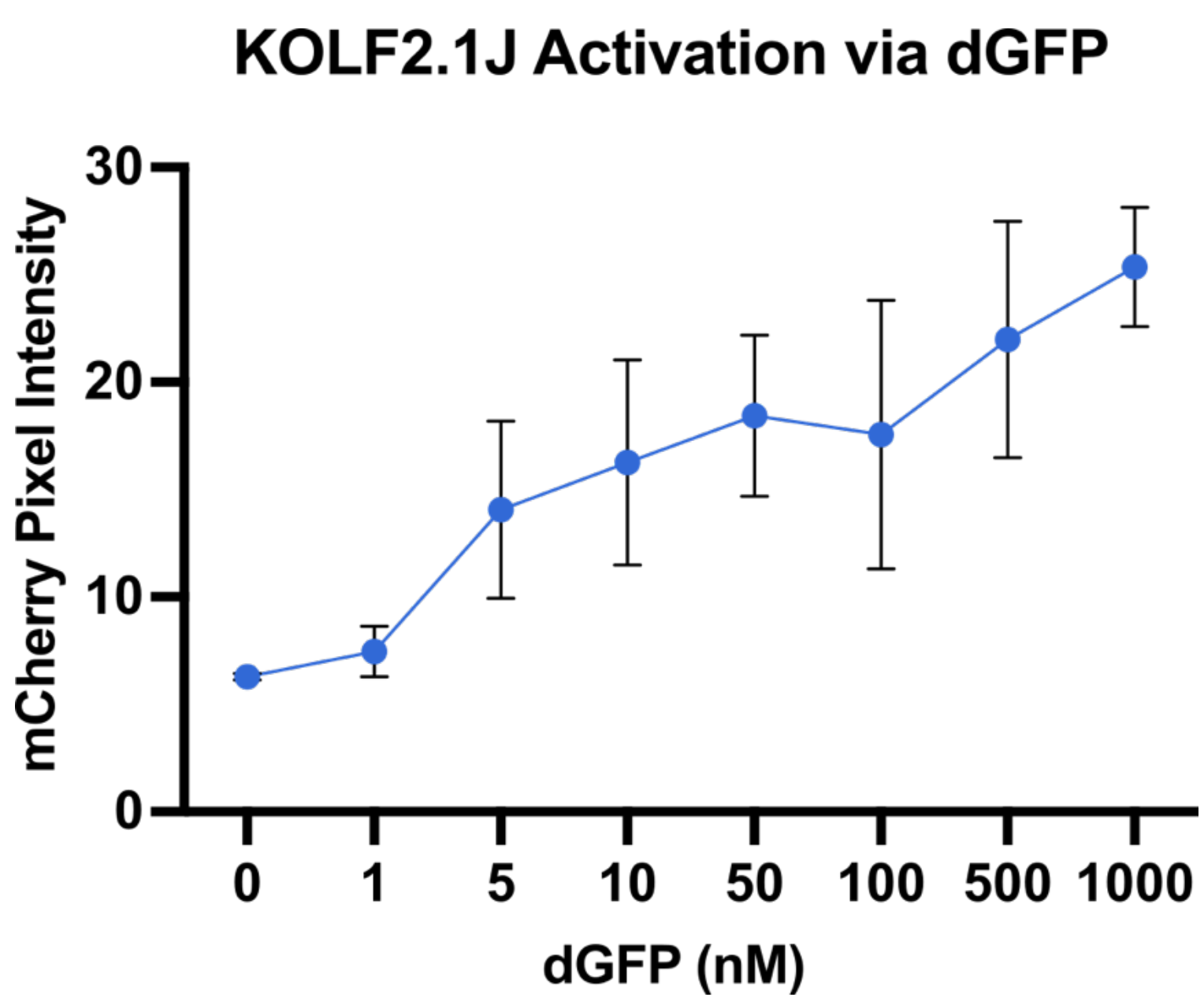

Supplementary Figure 2. KOLF2.1J iPSC dimeric superfolder GFP (dGFP) activation. dGFP dose response in KOLF 2.1J human induced pluripotent stem cells (iPSCs) engineered with a GFP-responsive LaG16 synNotch receptor and an inducible mCherry fluorescent protein payload. Cells were activated with 0-1000nM of dGFP for 48 hours before being fixed and immunolabeled for mCherry expression and the nuclear marker DAPI. Cells were imaged and mean mCherry pixel intensity was quantified using ImageJ software. Error bars indicate SEM. *n* = 15, 5 images from 3 individual wells.

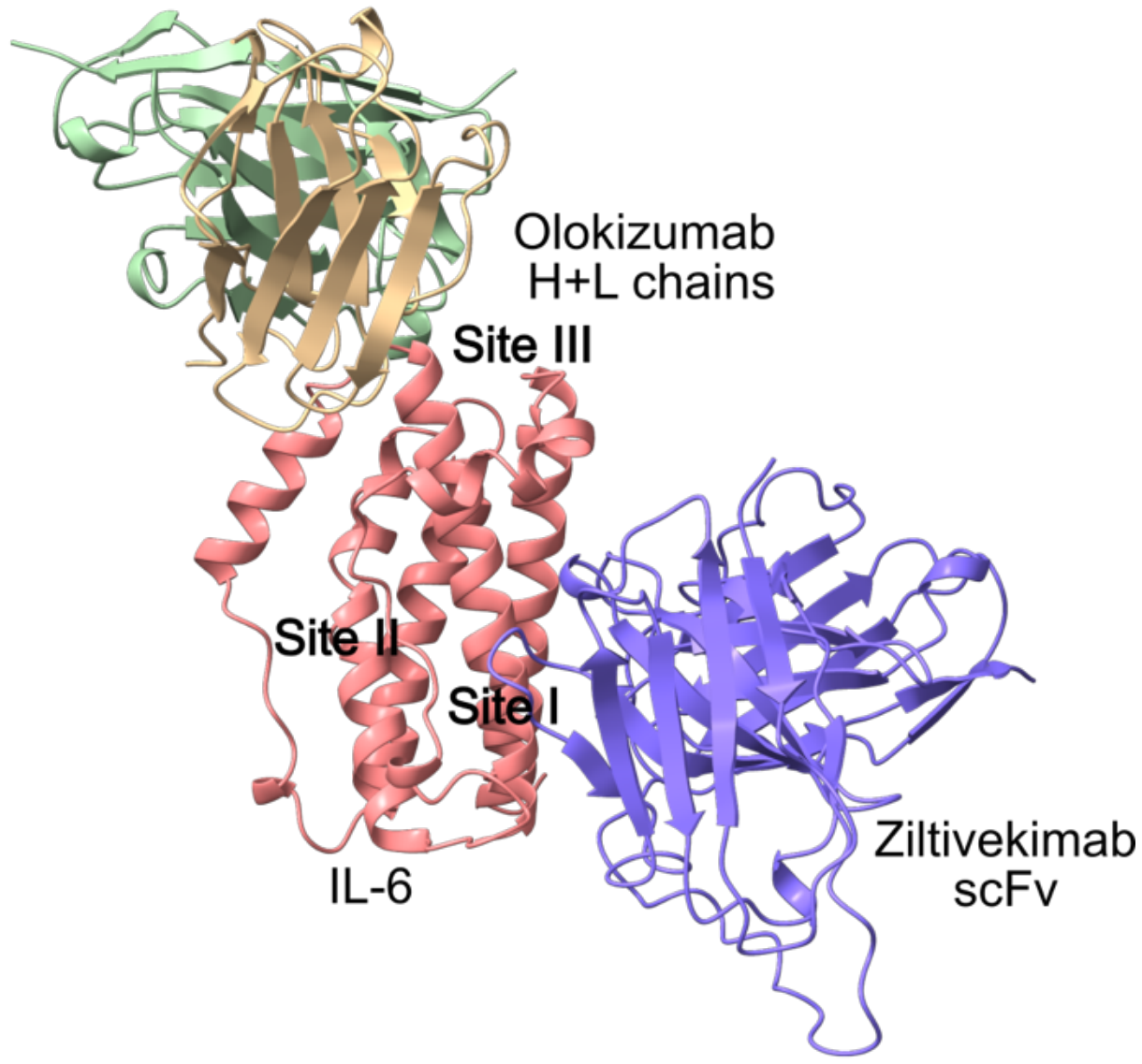

Supplementary Figure 3. Alphafold Multimer model of dual epitope binding of olokizumab and ziltivekimab variable chains to IL-6. AlphaFold multimer[9–15] modeling of IL-6 (Red) with ziltivekimab scFv (Blue)and olokizumab heavy (Green) and light (yellow) chains. Graphic demonstrates predicted ziltivekimab binding to site I of IL-6 as hypothesized and olokizumab binding to site III of IL-6 as described previously.[16,17] Model quality scores: pLDDT=91.7 pTM=0.844 ipTM=0.808

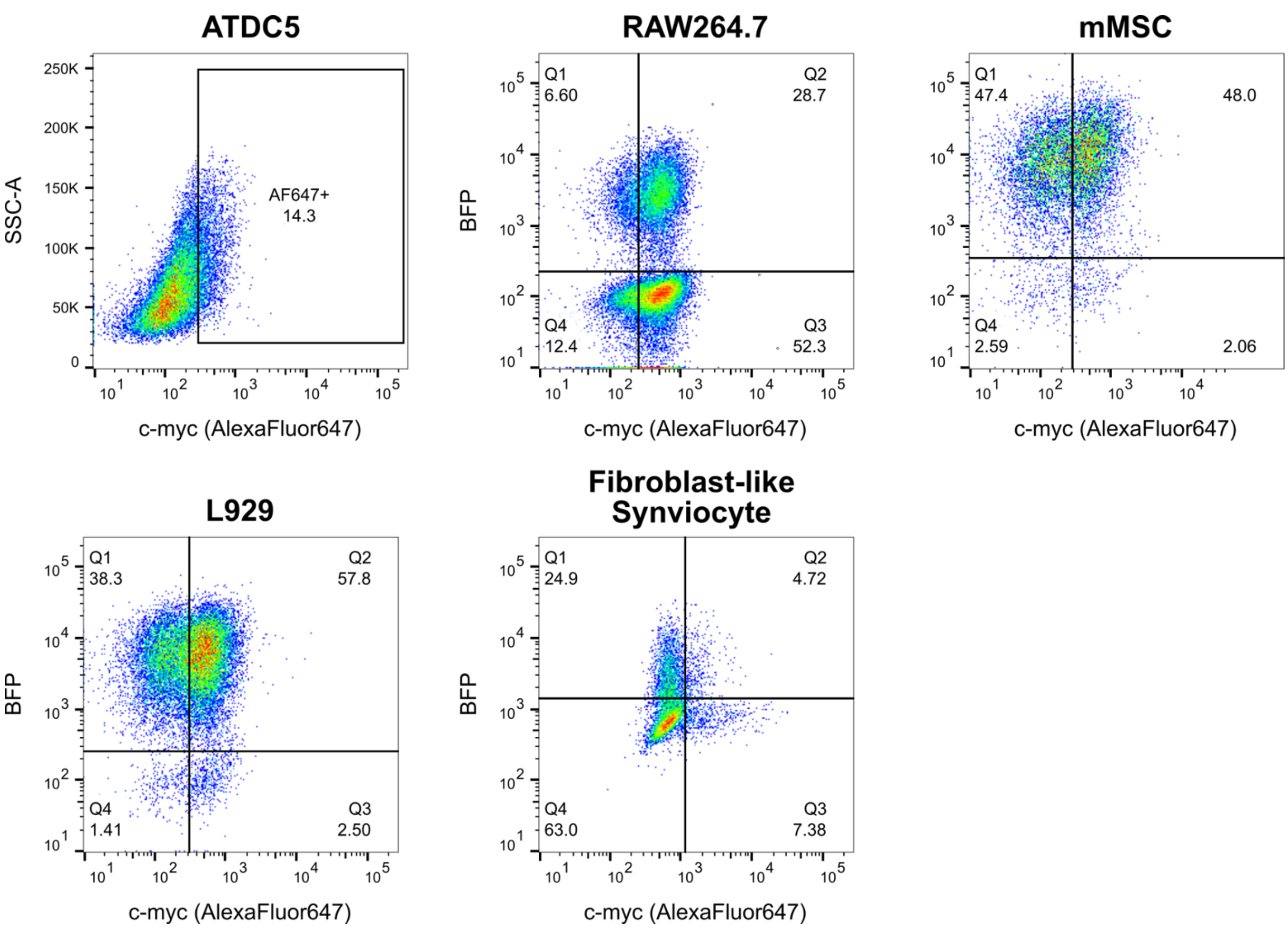

Supplementary Figure 4. Canak-Notch lentiviral transduction efficiency in cell types tested as measured by flow cytometry. Side scatter (ATDC5) or constitutive BFP from reporter circuit (all others) vs. c-myc. c-myc (x-axis) indicates expression of synNotch receptor while BFP indicates reporter expression. Cells above were selected with puromycin prior to flow cytometry, with the exception of fibroblast-like synoviocytes.

Supplementary Figure 5 (caption follows final plot)

Wild-type mMSC

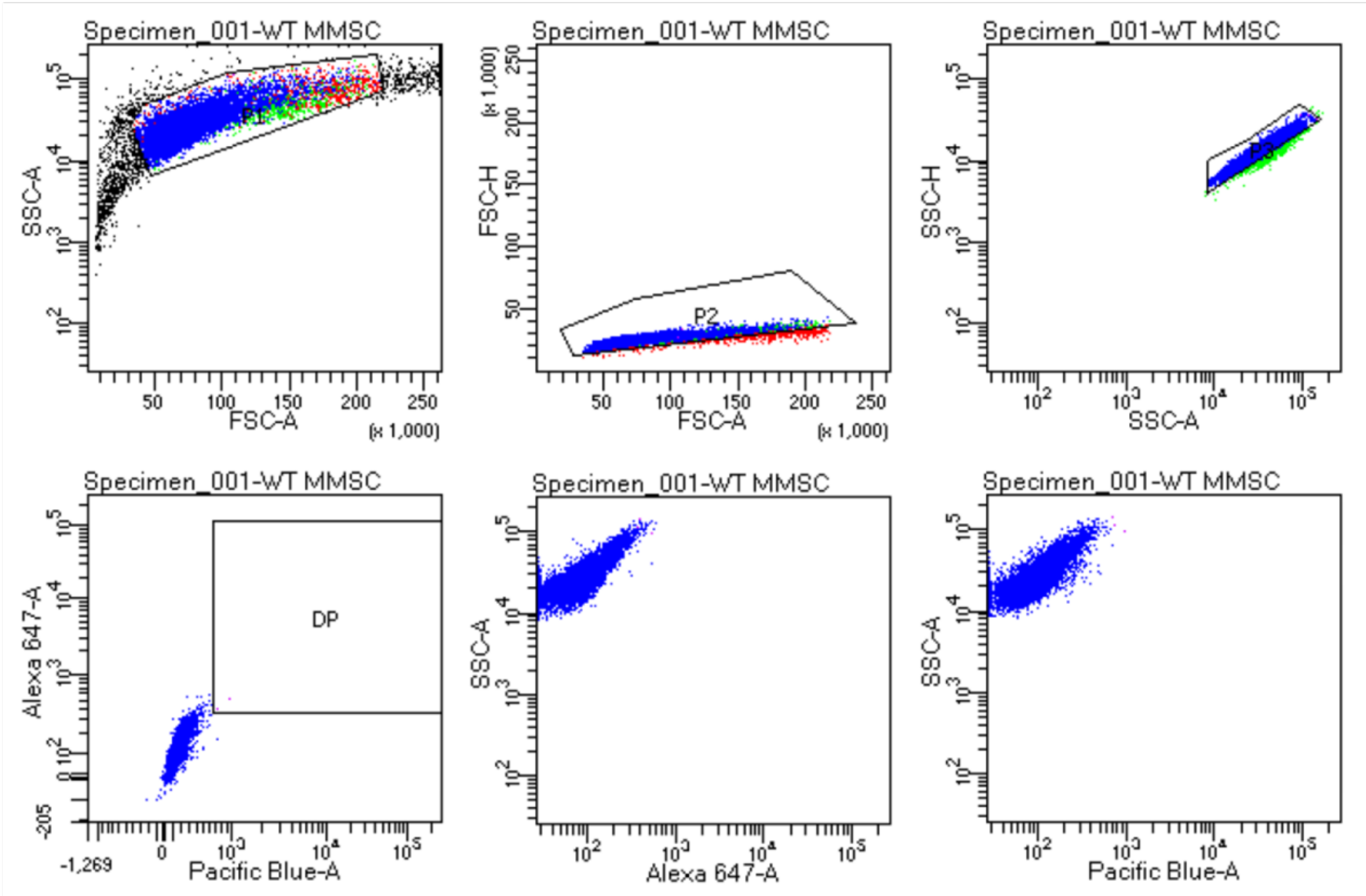

Canak-Notch mMSC

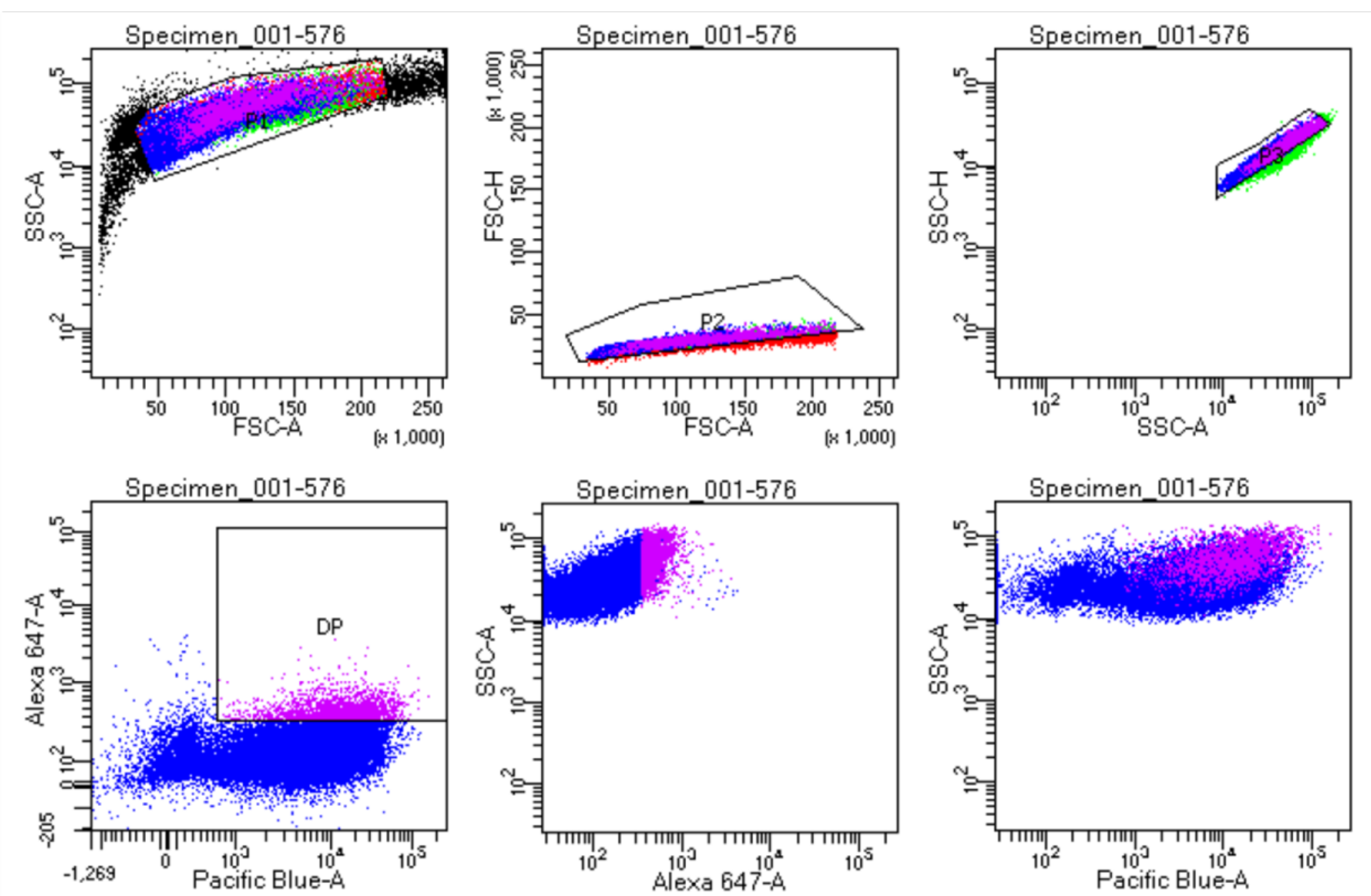

Canak-SNIPR mMSC

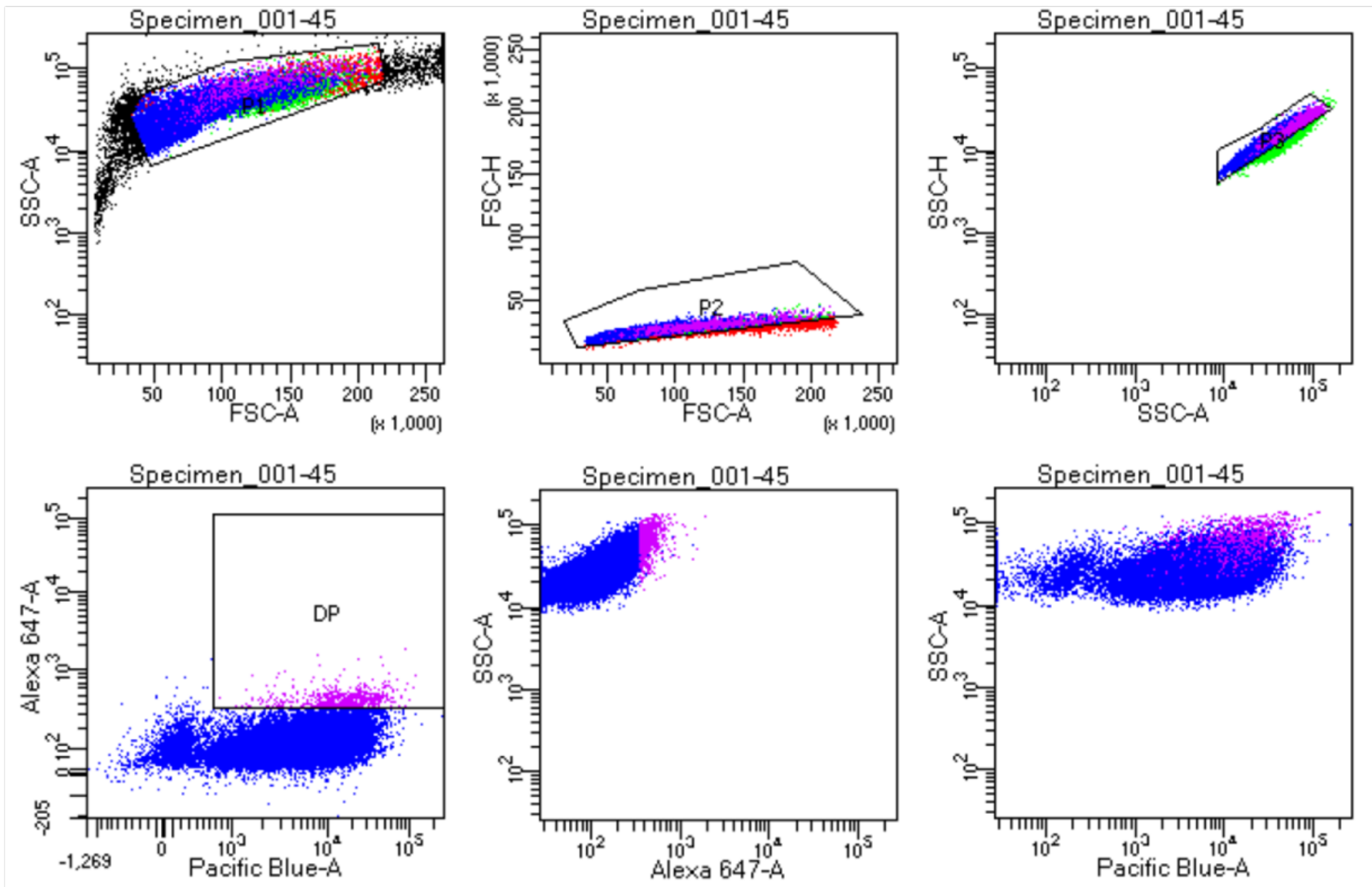

Zilti-Notch mMSC

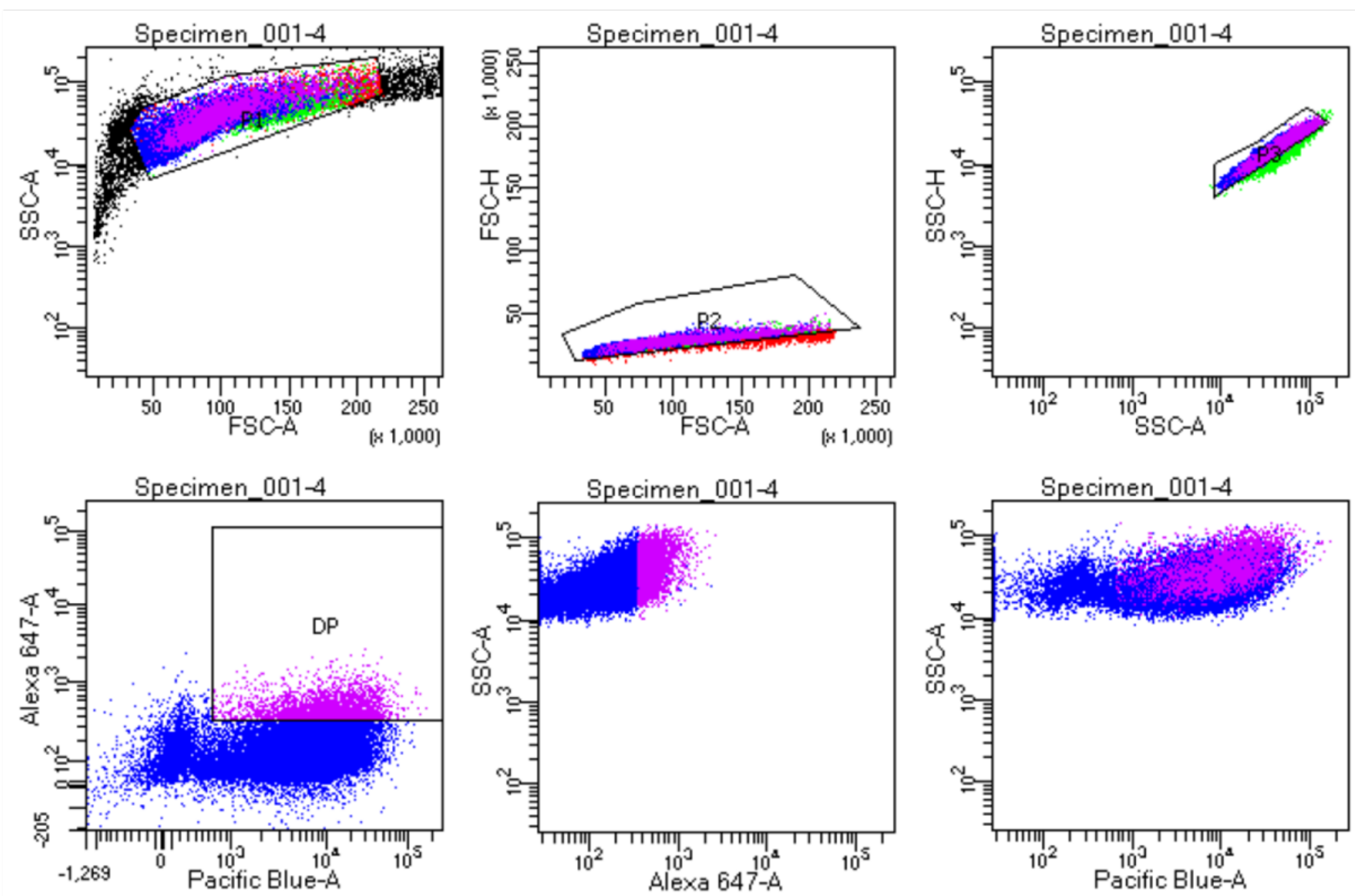

Zilti-SNIPR mMSC

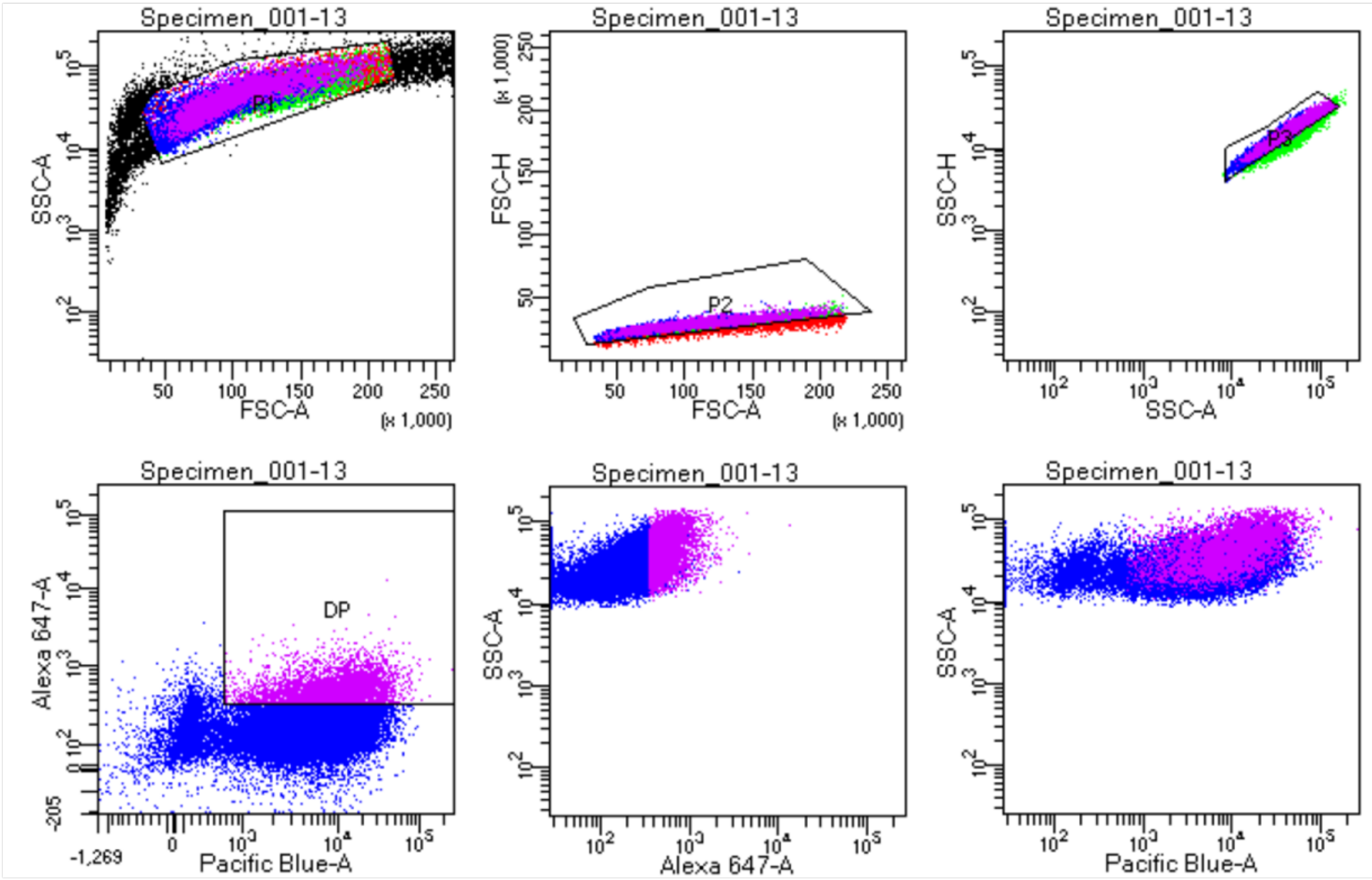

Supplementary Figure 5. Fluorescence activated cell sorting plots and gates for Figure 4. Alexa 647 indicates receptor expression detected via immunolabeling of an N-terminal c-myc epitope tag, and Pacific Blue indicates payload cassette expression via constitutive tagBFP expression. Plots show similar levels of expression between receptor constructs.

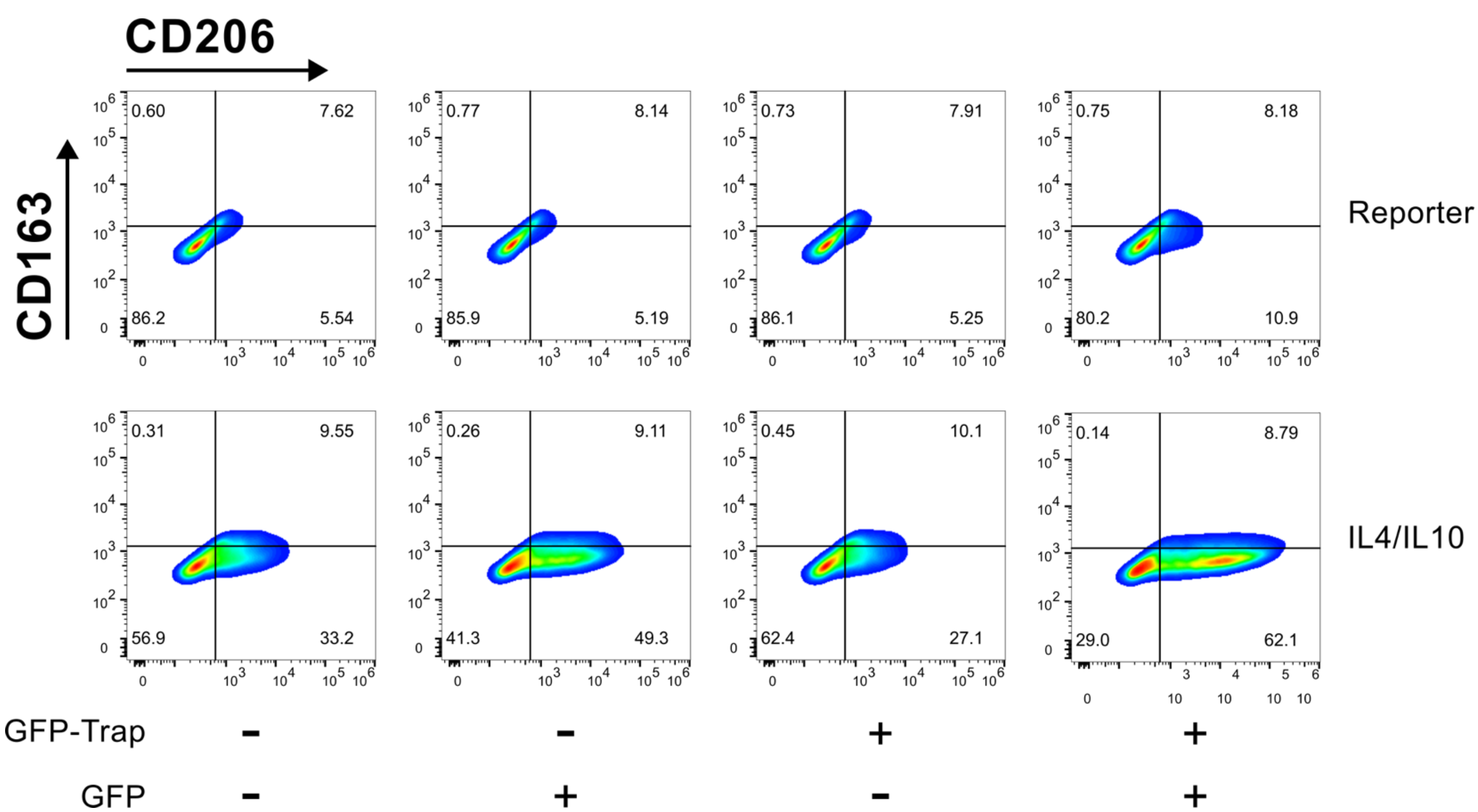

Supplementary Figure 6. Flow cytometry plots displaying RAW264.7 surface marker expression in response to treatment with conditioned media from GFP-responsive LaG16-Notch MSCs**.**

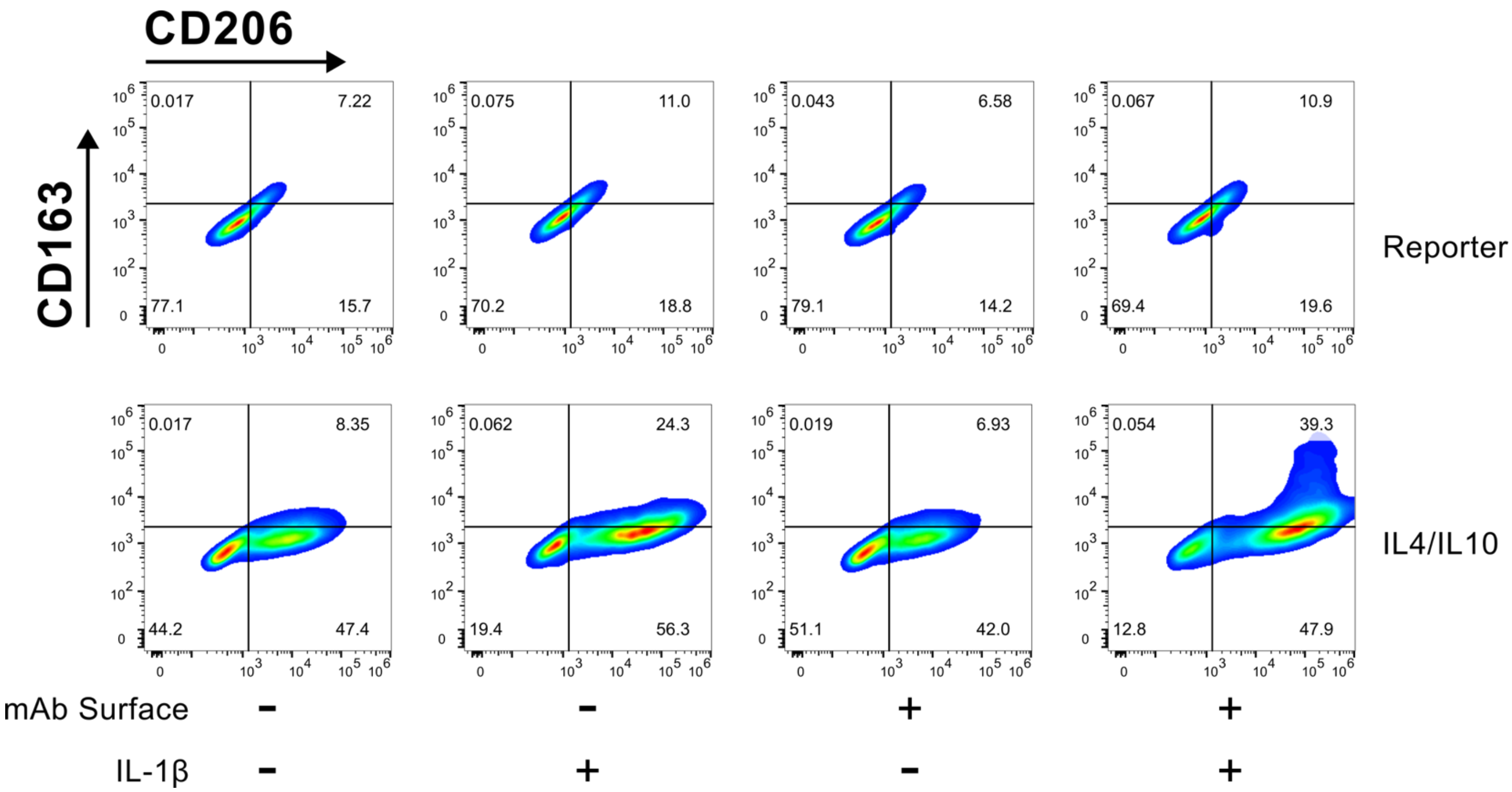

Supplementary Figure 7. Flow cytometry plots displaying RAW264.7 surface marker expression in response to co-culture with Canak-Notch MSCs and treatment with IL-1β.

Supplemental References:

[1] C.B. Pantazis, A. Yang, E. Lara, J.A. McDonough, C. Blauwendraat, L. Peng, H. Oguro, J. Kanaujiya, J. Zou, D. Sebesta, G. Pratt, E. Cross, J. Blockwick, P. Buxton, L. Kinner-Bibeau, C. Medura, C. Tompkins, S. Hughes, M. Santiana, F. Faghri, M.A. Nalls, D. Vitale, S. Ballard, Y.A. Qi, D.M. Ramos, K.M. Anderson, J. Stadler, P. Narayan, J. Papademetriou, L. Reilly, M.P. Nelson, S. Aggarwal, L.U. Rosen, P. Kirwan, V. Pisupati, S.L. Coon, S.W. Scholz, T. Priebe, M. Öttl, J. Dong, M. Meijer, L.J.M. Janssen, V.S. Lourenco, R. van der Kant, D. Crusius, D. Paquet, A.-C. Raulin, G. Bu, A. Held, B.J. Wainger, R.M.C. Gabriele, J.M. Casey, S. Wray, D. Abu-Bonsrah, C.L. Parish, M.S. Beccari, D.W. Cleveland, E. Li, I.V.L. Rose, M. Kampmann, C. Calatayud Aristoy, P. Verstreken, L. Heinrich, M.Y. Chen, B. Schüle, D. Dou, E.L.F. Holzbaur, M.C. Zanellati, R. Basundra, M. Deshmukh, S. Cohen, R. Khanna, M. Raman, Z.S. Nevin, M. Matia, J. Van Lent, V. Timmerman, B.R. Conklin, K. Johnson Chase, K. Zhang, S. Funes, D.A. Bosco, L. Erlebach, M. Welzer, D. Kronenberg-Versteeg, G. Lyu, E. Arenas, E. Coccia, L. Sarrafha, T. Ahfeldt, J.C. Marioni, W.C. Skarnes, M.R. Cookson, M.E. Ward, F.T. Merkle, A reference human induced pluripotent stem cell line for large-scale collaborative studies, Cell Stem Cell 29 (2022) 1685-1702.e22. https://doi.org/10.1016/j.stem.2022.11.004.

[2] K. Silence, M.J. Lauwereys, T. Dreier, Stabilized single domain antibodies, US20170107302A1, 2017. https://patents.google.com/patent/US20170107302A1/en (accessed August 27, 2025).

[3] S.R. Ledbetter, C.P. Hart, R.G. Holgate, L.U. Jermutus, C.L. Buchanan, A.R. Duncan, D.K. Finch, Antibodies to tgf-beta, US20140127230A1, 2014. https://patents.google.com/patent/US20140127230A1/en (accessed March 13, 2025).

[4] K.A. Schwarz, N.M. Daringer, T.B. Dolberg, J.N. Leonard, Rewiring human cellular input–output using modular extracellular sensors, Nat Chem Biol 13 (2017) 202–209. https://doi.org/10.1038/nchembio.2253.

[5] W.-C. Liang, X. Wu, F.V. Peale, C.V. Lee, Y.G. Meng, J. Gutierrez, L. Fu, A.K. Malik, H.-P. Gerber, N. Ferrara, G. Fuh, Cross-species Vascular Endothelial Growth Factor (VEGF)-blocking Antibodies Completely Inhibit the Growth of Human Tumor Xenografts and Measure the Contribution of Stromal VEGF*, Journal of Biological Chemistry 281 (2006) 951–961. https://doi.org/10.1074/jbc.M508199200.

[6] M. Blech, D. Peter, P. Fischer, M.M.T. Bauer, M. Hafner, M. Zeeb, H. Nar, One Target—Two Different Binding Modes: Structural Insights into Gevokizumab and Canakinumab Interactions to Interleukin-1β, Journal of Molecular Biology 425 (2013) 94–111. https://doi.org/10.1016/j.jmb.2012.09.021.

[7] H. Zhong, A. Davis, M. Ouzounova, R.A. Carrasco, C. Chen, S. Breen, Y.S. Chang, J. Huang, Z. Liu, Y. Yao, E. Hurt, J. Moisan, M. Fung, D.A. Tice, S.G. Clouthier, Z. Xiao, M.S. Wicha, H. Korkaya, R.E. Hollingsworth, A Novel IL6 Antibody Sensitizes Multiple Tumor Types to Chemotherapy Including Trastuzumab-Resistant Tumors, Cancer Res 76 (2016) 480–490. https://doi.org/10.1158/0008-5472.CAN-15-0883.
[8] S. Shaw, T. Bourne, C. Meier, B. Carrington, R. Gelinas, A. Henry, A. Popplewell, R. Adams, T. Baker, S. Rapecki, D. Marshall, A. Moore, H. Neale, A. Lawson, Discovery and characterization of olokizumab, MAbs 6 (2014) 773–781. https://doi.org/10.4161/mabs.28612.
[9] H. Berman, K. Henrick, H. Nakamura, Announcing the worldwide Protein Data Bank, Nat. Struct. Biol. 10 (2003) 980. https://doi.org/10.1038/nsb1203-980.
[10] R. Evans, M. O'Neill, A. Pritzel, N. Antropova, A. Senior, T. Green, A. Zidek, R. Bates, S. Blackwell, J. Yim, O. Ronneberger, S. Bodenstein, M. Zielinski, A. Bridgland, A. Potapenko, A. Cowie, K. Tunyasuvunakool, R. Jain, E. Clancy, P. Kohli, J. Jumper, D. Hassabis, Protein complex prediction with AlphaFold-Multimer, bioRxiv (2021). https://doi.org/10.1101/2021.10.04.463034v1.
[11] M. Mirdita, K. Schütze, Y. Moriwaki, L. Heo, S. Ovchinnikov, M. Steinegger, ColabFold: Making Protein folding accessible to all, Nature Methods (2022). https://doi.org/10.1038/s41592-022-01488-1.
[12] M. Mirdita, M. Steinegger, J. S"oding, MMseqs2 desktop and local web server app for fast, interactive sequence searches, Bioinformatics 35 (2019) 2856–2858. https://doi.org/10.1093/bioinformatics/bty1057.
[13] M. Mirdita, L. von den Driesch, C. Galiez, M.J. Martin, J. S"oding, M. Steinegger, Uniclust databases of clustered and deeply annotated protein sequences and alignments, Nucleic Acids Res. 45 (2017) D170–D176. https://doi.org/10.1093/nar/gkw1081.
[14] M. Steinegger, M. Meier, M. Mirdita, H. V"ohringer, S.J. Haunsberger, J. S"oding, HH-suite3 for fast remote homology detection and deep protein annotation, BMC Bioinform. 20 (2019) 473. https://doi.org/10.1186/s12859-019-3019-7.
[15] M. van Kempen, S.S. Kim, C. Tumescheit, M. Mirdita, J. Lee, C.L.M. Gilchrist, J. S"oding, M. Steinegger, Fast and accurate protein structure search with Foldseek, Nature Biotechnology (2023). https://doi.org/10.1038/s41587-023-01773-0.
[16] R.D. Metcalfe, T.L. Putoczki, M.D.W. Griffin, Structural Understanding of Interleukin 6 Family Cytokine Signaling and Targeted Therapies: Focus on Interleukin 11, Front Immunol 11 (2020) 1424. https://doi.org/10.3389/fimmu.2020.01424.
[17] Tocilizumab: An Updated Review of Its Use in the Treatment of Rheumatoid Arthritis and Its Application for Other Immune-Mediated Diseases - Toshio Tanaka, Atsushi Ogata, Masashi Narazaki, 2013, (n.d.). https://journals.sagepub.com/doi/10.4137/CMT.S9282 (accessed August 29, 2025).
